# Regioselective Synthesis and Cytotoxic Effects of New Juglone Derivatives with an Aliphatic Substituent at C(2) or C(3)

**DOI:** 10.3390/biom15121708

**Published:** 2025-12-06

**Authors:** Giovanni Vidari, Emanuele Casali, Andrea Magni, Sirwan T. Othman, Giuseppe Zanoni, Alessio Porta

**Affiliations:** 1 Dipartimento di Chimica, Università degli Studi di Pavia, Via Taramelli, 12-27100 Pavia, Italy; emanuele.casali@unipv.it (E.C.); andrea.magni@unipv.it (A.M.); sirwan.othman@su.edu.krd (S.T.O.); gzanoni@unipv.it (G.Z.); 2 Department of Medical Analysis, Faculty of Applied Science, Tishk International University, Erbil 44001, Iraq; 3 Department of Chemistry, College of Science, Salahaddin University-Erbil, Erbil 44002, Iraq

**Keywords:** organic synthesis, naphthoquinones, 2-substituted juglone derivatives, MTT test, in silico study

## Abstract

The naphthoquinone juglone (5-hydroxynaphthalene-1,4-dione) (**1**) occurs abundantly in nature, especially in species belonging to the *Juglandaceae* family. Due to its multifaceted biological activities, this compound is considered a privileged structure in Medicinal Chemistry for the development of new prototypes with several biological and pharmacological actions. However, the regioselective synthesis of 2-substituted juglones is challenging due to the non-symmetric naphthoquinone nucleus. Starting from non-symmetric 2,3-unsubstituted naphthalenes, in this paper we describe two general synthetic routes to juglone derivatives bearing an unsaturated or an oxygenated aliphatic side chain at C(2) or C(3). In an MTT test, a few products were more active than the parent unsubstituted juglone as inhibitors of the viability of human lung cancer H460 and breast cancer MCF-7 cells. The most potent compound featured a 1′-acetoxyhomoprenyl sidechain at the carbon C(2) of juglone.

## 1. Introduction

**Figure 1:**
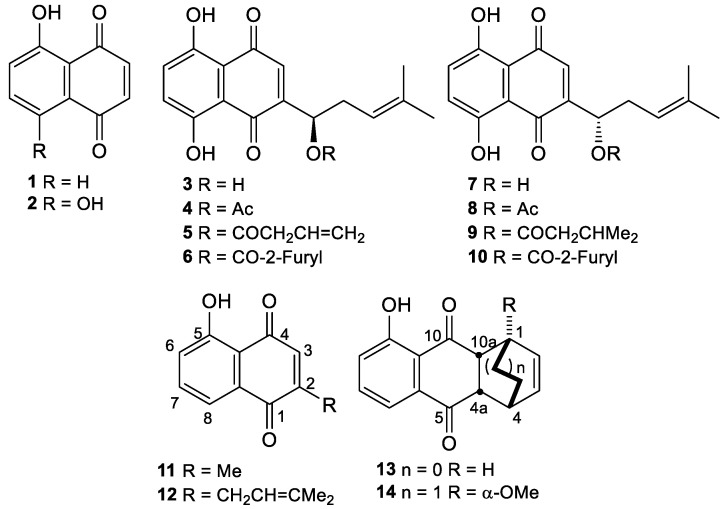
Structures of naturally occurring juglone (**1**), naphthazarin (**2**), shikonin (**3**), alkannin (**7**), plumbagin (**11**), and a few synthetic derivatives (**4**–**6**, **8**–**10**, **12**, **13**, **14**) reported in the literature.

The 1,4-naphthoquinone juglone (5-hydroxynaphthalene-1,4-dione) (**1**) (Figure 1) occurs abundantly in nature, especially in several *Juglans* species (Juglandaceae) [1,2,3]. In addition to having sedative, antioxidant, antihypertensive, antifungal, antibacterial, antiviral, and allelopathic properties, this naphthoquinone and some derivatives have emerged as promising compounds in cancer research due to their multifaceted cytotoxic effects and action mechanisms on different tumors, including the inhibition of cell viability and proliferation, induction of autophagy and DNA damage, angiogenesis inhibition, regulation of cell death pathways such as ferroptosis and apoptosis, and disruption of metastatic processes [3,4,5,6,7,8,9]. Moreover, juglone was found to block RNA polymerases II and III, human Pin1 [3], and *E. coli* parvulin and the yeast Ess1/Ptf1; it inhibits aromatase cytochrome P450 and topoisomerase I with an activity level (MIC = 5 μM) ten times higher than that of the reference compound naphthazarin (**2**) (MIC = 50 μM) [10]. In conclusion, its large availability and wide spectrum of biological activities make juglone a privileged structure in Medicinal Chemistry for the development of new prototypes with various biological and pharmacological actions [1,3].

It is worth noting that several naphthoquinones bearing aliphatic substituents exhibit biological activities more potent than those of unsubstituted parent compounds [11]. In a paradigmatic example, a few years ago, the research groups of Ahn and Couladouros examined the in vitro activity against topoisomerase 1 of shikonin (**3**) and alkannin (**7**) (Figure 1), which are substituted at C(2) by an enantiomeric 1–hydroxyhomoprenyl side chain. Shikonin (**3**) retained the moderate activity of the unsubstituted parent compound, naphthazarin (**2**) [10,12,13,14], and was ten times more active than compound **7** (Figure 1) [8]. However, acylation of the hydroxyl group of compounds **3** and **7** with unsaturated or saturated short chain (C_2_-C_6_) acids, to give esters **4**–**6** and **8**–**10**, respectively, increased the activity against topoisomerase I significantly and independently of the carbinol absolute configuration [10,15]. Thus, alkannin isopentanoate **9** was a hundred times more potent in vitro than the corresponding free alcohol **7** [10], while shikonin esters **4** and **5** were three times more potent than the well-known topoisomerase inhibitor camptothecin [13].

Inspired by the high biological activity exhibited by 2-substituted naphthazarin derivatives, especially by shikonin (**3**) and its derivatives, we studied the regioselective synthesis of a few novel juglone derivatives bearing an unsaturated or an oxygenated short (C_3_-C_5_) side chain at C(2) or C(3) (juglone numbering system) of the naphthoquinone nucleus. We were conscious that the regioselective synthesis of non-symmetric juglone derivatives was more challenging than the substitution of the highly symmetric naphthazarin nucleus (**2**) [13]; therefore, most synthetic approaches to substituted naphthazarin derivatives [14,16] could not be extended to juglone derivatives. In this context, we were aware of only two regioselective synthetic routes to monosubstituted 2-alkyl juglones, i.e., plumbagin (2-methyljuglone, **11**) and 2-prenyl juglone (**12**), that required, however, the intermediate preparation of the Diels-Alder adducts **13** and **14**, respectively [17,18].

**Figure 2:**
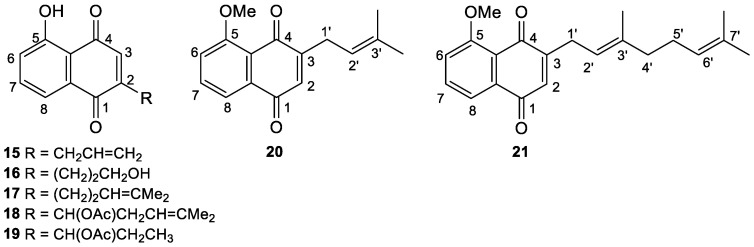
Novel 2- and 3-substituted juglone derivatives synthesized in this work.

In this paper, starting from 2,3-unsubstituted naphthoquinones, we accomplished the highly regioselective synthesis of the well-known 2-prenyl juglone **12**; five new 2-substituted juglones, **15**, **16**, **17**, **18**, and **19**; and two new 3-substituted *O*-methyl juglones, **20** and **21** (Figure 2). Moreover, the inhibitory effects of synthesized compounds on the viability of human lung cancer H460 and breast cancer MCF-7 cells were determined with an in vitro MTT test [19].

## 2. Materials and Methods

### 2.1. Chemicals and Instruments

Solvents, chemical reagents, biochemical material, TLC plates and silica gel powder for preparative column chromatography, juglone (**1**) and shikonin (**3**) were purchased from Merck/Sigma-Aldrich (Milano (MI), Italy); 1,4,5-trimethoxy-naphthalene (**20**) was purchased from RR Scientific (Irwindale, CA, USA; http://www.rrscientific.com). High-resolution mass spectra (HRMS) were obtained with a Finnigan-MAT TSQ-70 (Finnigan Corporation, Thermo Scientific, Waltham, MA, USA) spectrometer. 1D and 2D NMR spectra were determined on a Bruker Avance III 300 MHz NMR instrument (Bruker Italia Srl, Milano (MI), Italy) at 25 °C. Nuclear Overhauser effects were determined with monodimensional NOE difference spectra. Flash column chromatographic separations were performed using Merck Silica gel 60 (0.015–0.040 mm); TLC and PLC separations were carried out on Merck HF_254_ silica gel plates. The purity of products was checked by TLC, NMR and MS and deemed sufficient for the purpose of structural determination. Melting points were measured on a Fisher-Johns hot plate apparatus (Fischer Scientific Italia, 20054 Segrate (MI), Italy) and were uncorrected. Computational studies were carried out with Gaussian 09D (for DFT calculations) and docking calculations with Maestro by Schrödinger (LLC, New York, NY, USA, 2025).

### 2.2. General Information

All solvents were of commercial quality and were purified by distillation over the drying agents indicated: THF (Na/benzophenone); dichloromethane (DCM), hexane, pyridine (CaH_2_); toluene (Na/K). All other reagents were used as supplied. All moisture-sensitive reactions were carried out under a positive static atmosphere of Ar in flame-dried glassware. Syringes and needles for the transfer of reagents were dried at 140 °C and allowed to cool in a desiccator over P_2_O_5_ before use. Reactions were monitored using silica gel 60 (0.25 mm), aluminum-supported TLC plates. Compounds were visualized under UV light at a wavelength of 254 nm or stained by spraying TLC plates with a 0.5% solution of vanillin in H_2_SO_4_/EtOH, followed by heating on a hot plate. Yields are reported for isolated compounds with >95% purity established by NMR. Chemical shifts (δ) are reported in ppm, coupling constants (*J*) in Hz. Chloroform signals were used as references and the chemical shifts converted to the TMS scale (*C*DCl_3_: δ_C_ 77.00; residual C*H*Cl_3_ in CDCl_3_: δ_H_ 7.26). COSY, DEPT, HSQC, and NOESY spectra were recorded using standard pulse sequences.

### 2.3. General Synthetic Procedures

#### 2.3.1. CAN Oxidation: Representative Example

Ceric (IV) ammonium nitrate, (NH_4_)_2_ [Ce(NO_3_)_6_] (CAN, 144 mg, 0.26 mol, 2 equiv), was added to a solution of compound **33** (16.7 mg, 0.065 mmol) in MeCN/H_2_O, 9:1 (2 mL, 0.0134 mL/mg). The resulting mixture was stirred at 22 °C for 1.5 h; then the reaction was quenched by adding brine (5 mL) and DCM (15 mL); the organic layer was separated, and the aqueous phase was extracted with DCM (3 × 4 mL). The combined organic layers were dried over MgSO_4_ and evaporated under vacuum. The resulting residue was separated on silica gel (10 g). Elution with hexane-EtOAc, 9:1, afforded 2-allyljuglone (**15**, 9.9 mg, yield = 71%).

The same procedure was followed for the oxidation of compounds **26**, **27**, **39**, **41**, **42**, **43**, and **44** acetate to **20**, **21**, **18**, **12**, **16**, **17**, and **19**, respectively.

#### 2.3.2. Metathesis Reaction: Representative Example

2nd Generation Grubbs catalyst **37** [20] (14.4 mg, 0.017 mmol, 0.1 equiv) was added to a solution of homoallyl alcohol **36** (52 mg, 0.17 mmol) in DCM (3 mL) containing excess 2-methyl-2-butene (20 equiv) under Ar in a sealed vial. The reaction mixture was heated to 55 °C for 16 h. Volatiles were then removed, and the resulting residue was separated on a silica gel column (10 g). Elution with hexane/EtOAc, 9:1, afforded alcohol **38** (45.5 mg, yield = 80%).

The same procedure was followed for the conversion of compound **40** into **41**.

### 2.4. 5-Hydroxy-2-(3-methylbut-2-en-1-yl)naphthalene-1,4-dione (***12***)

Compound **12** (yield = 70% from **41**, following the procedure described in Section 2.3.1). HRMS (ESI positive mode) C_15_H_15_O_3_ [M + H]^+^ calc. 243.1021, found 243.1018. IR and ^1^H-NMR spectra matched those reported in the literature [16]. ^13^C-NMR (75 MHz, CDCl_3_) δ 189.1, 184.3, 161.8, 146.4, 136.9, 135.3, 135.1, 133.4, 124.3, 120.1, 119.9, 115.9, 27.7, 25.7, 19.8.

### 2.5. 2-Allyl-5-hydroxynaphthalene-1,4-dione (***15***)

Compound **15** (yield = 71% from **33**, following the general procedure described in Section 2.3.1): dark yellow/brown crystals, mp 165–168 (dec.); HRMS (ESI positive mode) C_13_H_11_O_3_^+^ [M + H]^+^ calc. 215,071, found 215,073. ^1^H-NMR (300 MHz, CDCl_3_) δ_H_ 7.73 (1H, dd, *J* = 7.5, 1.4 Hz), 7.68–7.42 (2H, m), 6.46 (1H, t, *J* = 0.8 Hz, 3-H), 5.86 (1H, ddt, *J* = 16.8, 10.1, 7.6 Hz, 2′-H), 5.16 (1H, ddt, *J* = 16.9, 2.3, 1.5 Hz, 3′-H*_E_*), 4.95 (1H, ddt, *J* = 10.1, 2.2, 1.5 Hz, 3′-H*_Z_*), 3.35 (2H, dtd, *J* = 7.5, 1.5, 0.8 Hz, 1′-H_2_). ^13^C-NMR (75 MHz, CDCl_3_) δ_C_ 189.1, 184.1, 161.7, 156.1, 137.2, 135.7, 135.6, 133.4, 124.4, 119.9, 116.1, 115.9, 33.3.

### 2.6. 5-Hydroxy-2-(3-hydroxypropyl)naphthalene-1,4-dione (***16***)

Compound **16** (yield = 51% from **42**, following the general procedure described in Section 2.3.1): dark yellow/brown oil. HRMS (ESI positive mode) C_13_H_13_O_4_^+^ [M + H]^+^ calc. 233,0814, found 233,0809. ^1^H-NMR (300 MHz, CDCl_3_) δ_H_ 7.73 (1H, dd, *J* = 7.5, 1.4 Hz), 7.63 (1H, dd, *J* = 8.2, 7.5 Hz, 7-H), 7.50 (1H, dd, *J* = 8.2, 1.3 Hz, 1H), 6.07 (1H, t, *J* = 0.8 Hz, 3-H), 3.68 (1H, dd, *J* = 6.3, 4.9 Hz, OH), 3.64–3.47 (2H, m, 3′-H_2_), 2.85 (2H, td, *J* = 8.3, 0.9 Hz, 1′-H_2_), 1.86 (2H, tt, *J* = 8.3, 5.8 Hz, 2′-H_2_). ^13^C-NMR (75 MHz, CDCl_3_) δ_C_ 189.2, 186.5, 161.7, 149.0, 137.2, 135.3, 133.1, 124.3, 119.9, 115.8, 62.1, 31.3, 27.5.

### 2.7. 5-Hydroxy-2-(4-methylpent-3-en-1-yl)naphthalene-1,4-dione (***17***)

Compound **17** (yield = 48% from **43**, following the general procedure described in Section 2.3.1): dark yellow/brown oil. HRMS (ESI positive mode) C_16_H_17_O_3_^+^ [M + H]^+^ calc. 257.1178, found 257.1174.^1^H-NMR (300 MHz, CDCl_3_) δ*_H_* 11.94 (1H, s, OH), 7.65 (1H, d, *J* = 7.2 Hz, 8-H*), 7.63 (1H, d, *J* = 6.8 Hz, 6-H*), 7.29 (1H, dd, *J* = 7.2 and 6.8 Hz, 7-H), 6.78 (1H, s, 3-H), 5.19 (1H, br t, *J* = 7.2 Hz, 3′-H), 2.75 (2H, dd, *J* = 7.2 and 7.5 Hz, 1′-H_2_), 2.30 (2H, br q, *J* = 7.3 Hz, 2′-H_2_), 1.72 (3H, br s, 4′-Me*_E_*), 1.61 (3H, s, 4′-Me*_Z_*). * Assignments can be interchanged. ^13^C-NMR (75 MHz, CDCl_3_) δ*_C_* 189.2, 186.3, 161.7, 147.4, 137.2, 135.3, 133.1, 132.7, 124.3, 123.0, 119.9, 115.8, 29.0, 27.1, 25.5, 19.6.

### 2.8. 1-(5-Hydroxy-1,4-dioxo-1,4-dihydronaphthalen-2-yl)-4-methylpent-3-en-1-yl Acetate: (±)-***18*** and Resolution of the Racemic Mixture

Compound (±)-**18** (yield = 74% from acetate **39**, following the general procedure described in Section 2.3.1): dark yellow/brown crystals; mp 173–177 °C (dec.). HRMS (ESI positive mode) C_18_H_19_O_5_^+^ [M + H]^+^ calc. 315.1232, found 315.1228. ^1^H-NMR (300 MHz, CDCl_3_) (Figure S1 in the Supplementary Materials) δ_H_ 7.70–7.57 (2H, m, 8- and 7-H), 7.29 (1H, dd, *J* = 6.4, 3.2 Hz, 6-H), 6.82 (1H, br s, 3-H), 5.96 (1H, tdd, *J* = 6.5, 1.9, 0.7 Hz, 1′-H), 5.20–5.12 (1H, m, 3′-H), 2.69–2.52 (1H, m, 2′-H_a_), 2.50–2.32 (1H, m, 2′-H_b_), 2.16 (3H, s, *Me*CO), 1.70 and 1.59 (2 × 3H, 2 s, 4′-Me_2_). ^13^C-NMR (75 MHz, CDCl_3_) (Figure S2 in the Supplementary Materials) δ_C_ 189.1, 182.7, 169.7, 161.3, 150.5, 136.5, 136.1, 133.2, 131.9, 124.5, 119.4, 117.6, 114.8, 69.7, 32.7, 25.7, 20.9, 18.0.

Racemic **18** (5 mg) was separated (Figure S13 in the Supplementary Materials) into the two enantiomers **18a** and **18b** (unassigned absolute configuration) in 95% yield by HPLC resolution on a semi-preparative enantioselective ChiralPack AS-H column (Agilent Technologies Italia S.p.A., 20063 Cernusco sul Naviglio (MI), Italy); 250 × 2.1 mm; particle size: 5 mm; isocratic elution at 23 °C; eluent: *n*-heptane-isopropanol, 90:10; flow rate: 1 mL/min; UV detection at 254 nm.

### 2.9. 1-(5-Hydroxy-1,4-dioxo-1,4-dihydronaphthalen-2-yl) Propyl Acetate (***19***)

Pyridine (37.4 mg, 0.48 mmol) followed by Ac_2_O (49 mg, 0.48 mmol) and a catalytic amount of DMAP were added to a solution of alcohol **44** (69 mg, 0.24 mmol) in DCM (4 mL) cooled to 0 °C. The mixture was stirred overnight at 22 °C; subsequently, the reaction was quenched by addition of MeOH (50 μL), followed by saturated aqueous NaHCO_3_ (5 mL) and DCM (6 mL). The organic layer was separated, while the aqueous phase was extracted with an additional DCM (3 × 5 mL). The combined organic layers were washed with brine (3 mL), dried over Na_2_SO_4_, and evaporated. The resulting residue was separated on a silica gel column (10 g). Elution with hexane/EtOAc, 9:1, gave the desired acetate [δ_H_ 2.10 (3H, s, *Me*CO)] as a pale-yellow oil (75.3 mg, yield = 95%) which was used immediately in the next step. Then, CAN oxidative demethylation reaction, which was executed according to the general procedure outlined in 2.3.1, afforded compound **19** (30.9 mg, 49%) as a dark yellow/brown oil. HRMS (ESI positive mode) C_15_H_15_O_5_^+^ [M + H]^+^ calc. 275.0919, found 275.0922. ^1^H-NMR (300 MHz, CDCl_3_) δ_H_ 7.70–7.57 (2H, m, 6- and 8-H), 7.50 (1H, dd, *J* = 6.8, 2.7 Hz, 7-H), 6.86 (1H, s, 3-H), 5.93 (1H, ddqd, *J* = 6.3, 4.8, 1.5, 0.7 Hz, 1′-H), 2.10 (3H, s, *Me*CO), 1.88 (2H, m, 2′-H_2_), 1.00 (3H, td, *J* = 7.9, 1.5 Hz, 3′-H_3_). ^13^C-NMR (75 MHz, CDCl_3_) δ_C_ 186.9, 184.9, 169.9, 162.1, 143.3, 137.4, 132.4, 132.1, 123.3, 119.2, 114.4, 73.9, 26.8, 22.0, 11.6.

### 2.10. 3-(3′-Methyl-but-2′-enyl)-5-methoxy-naphthalene-1,4-dione (***20***)

By slightly modifying the general procedure described in Section 2.3.1, 3 equiv of CAN in MeCN-H_2_O (1:1, 2 mL) were used, and the reaction solution was extracted with EtOAc at the end of the reaction. Compound **20** (yield = 84% from **26**): sticky oil. EIMS *m/z* (rel. intensity): 256 (M^+^, 58), 241 (100), 213 (15). ^1^H-NMR (300 MHz, CDCl_3_) (Figure S4 in the Supplementary Materials) δ_H_ 7.72 (1H, dd, *J* = 7.6 and 1.3 Hz), 7.66 (1H, t, 8.0 Hz) and 7.30 (1H, d, 7.7 Hz) (6, 7- and 8-H), 6.70 (1H, t, *J* = 1.6 Hz, 2-H), 5.23 (1H, br t, *J* = 8.7 Hz, 2′-H), 4.02 (3H, s, 5-OMe), 3.25 (2H, br d, *J* = 7.3 Hz, 1′-H_2_), 1.78 and 1.66 (2 x 3H, 2 br s, 3′-Me_2_). ^13^C-NMR (75 MHz, CDCl_3_) (Figure S5 in the Supplementary Materials) δ_C_ 185.4, 184.6, 159.7, 152.6, 136.1, 134.7, 134.4, 132.5, 120.1, 118.7, 118.5, 117.6, 56.4, 28.2, 25.7, 17.8.

### 2.11. 3-(3′7-Dimethyl-octa-2,6-dienyl)-5-methoxy-naphthalene-1,4-dione (***21***)

Using the procedure described in Section 2.10, compound **27** was oxidized to **21** in 79% yield: sticky oil. EIMS *m/z* (rel. intensity): 324 (M^+^, 46), 255 (56), 241 (100), 223 (57). ^1^H-NMR (300 MHz, CDCl_3_) (Figure S7 in the Supplementary Materials) δ_H_ 7.74 (1H, dd, 7.6 and 1.3 Hz), 7.68 (1H, t, 8.0 Hz), and 7.32 (1H, dd, 8.3 and 1.3 Hz) (6, 7- and 8-H), 6.70 (1H, t, *J* = 1.3 Hz, 2-H), 5.25 and 5.12 (2 x 1H, 2 br t, 2′- and 6′-H), 4.03 (3H, s, 5-OMe), 3.28 (2H, br d, *J* = 7.3 Hz, 1′-H_2_), 2.4–2.1 (4H, m, 4′- and 5′-H_2_), 1.70, 1.63 and 1.60 (3 × 3H, 3 br s, 3′-Me and 7′-Me_2_). ^13^C-NMR (75 MHz, CDCl_3_) (Figure S8 in the Supplementary Materials) δ_C_ 185.4, 184.7, 159.7, 152.6, 139.8, 134.8, 134.4, 132.5, 131.8, 124.0, 120.2, 118.8, 118.4, 117.6, 56.4, 39.7, 28.0, 26.5, 25.7, 17.7, 16.1.

### 2.12. 1,4,5-Trimethoxy-3-(3′-methyl-but-2′-enyl)naphthalene (***26***)

1,4,5-Trimethoxy-naphthalene (**25**) (218 mg, 1.0 mmol, 1 equiv) in dry THF (5 mL) was cooled to 0 °C under argon and *n*-BuLi (2.5 M solution in hexane, 0.6 mL, 1.5 equiv) was added dropwise over 5 min. The mixture was stirred at 0 °C for 30 min; then prenyl bromide (268 mg, 1.8 mmol, 1.8 equiv) was added dropwise over 5 min. After stirring for 60 min at 22 °C, the mixture was poured into water and extracted with ether to give the crude product. Column chromatography (hexanes/EtOAc, 80:20) yielded the prenyl derivative **26** (200 mg, yield = 76%) accompanied by two unidentified regioisomers. EIMS *m/z* (rel. intensity): 286 (M^+^,100); 271 (39); 193 (10). ^1^H-NMR (300 MHz, CDCl_3_) δ_H_ 7.81, 7.31 and 6.88 3 × 1H, 3 m, 8-, 7- and 6-H), 6.65 (1H, s, 2-H), 5.35 (1H, m, 2′-H), 3.99 (3H, s, 5-OMe), 3.95 (3H, s, 1-OMe), 3.84 (3H, s, 4-OMe), 3.53 (2H, br d, *J* = 7.1 Hz, 1′-H_2_), 1.79 and 1.74 (2 × 3H, br s, 3′-Me_2_). ^13^C-NMR (75 MHz, CDCl_3_) δ_C_ 18.0, 25.8, 28.8, 55.8, 56.2, 62.3, 106.7, 106.9, 114.9, 120.8, 123.6, 124.8, 127.7, 130.8, 132.3, 146.9, 151.8, 155.5.

### 2.13. 1,4,5-Trimethoxy-3-(3′,7′-dimethyl-octa-2′,6′-dienyl)naphthalene (***27***)

Compound **27** (195 mg, yield = 55%), chromatographically separated from unidentified regioisomers, was obtained from 1,4,5-trimethoxy-naphthalene (**25**) (218 mg, 1.0 mmol, 1 equiv) with the procedure described for the preparation of compound **26**, using geranyl bromide (391 mg, 1.8 mmol) instead of prenyl bromide in the alkenylation step. EIMS *m/z* (rel. intensity): 354 (M^+^,100), 255 (20), 217 (10). ^1^H-NMR (300 MHz, CDCl_3_) δ_H_ 7.83, 7.32 and 6.89 (3 × 1H, 3 m, 8-, 7- and 6-H), 6.67 (1H, s, 2-H), 5.35 and 5.11 (2H, m, 2′- and 6′-H), 4.00 (3H, s, 5-OMe), 3.94 (3H, s, 1-OMe), 3.77 (3H, s, 4-OMe), 3.56 (2H, br d, *J* = 7.1 Hz, 1′-H_2_), 2.2–1.9 (4H, m, 4′- and 5′-H_2_), 1.79 (3H, br s, 3′-Me), 1.66 and 1.60 (2 × 3H, 2 br s, 7′-Me_2_). ^13^C-NMR (75 MHz, CDCl_3_) δ_C_ 154.3, 153.1, 151.2, 137.5, 131.7, 127.5, 125.8, 124.4, 124.3, 122.3, 119.8, 116.5, 110.2, 108.2, 61.3, 56.5, 55.9, 39.6, 28.8, 26.7, 25.0, 19.9, 16.3. 7

### 2.14. 6-(Allyloxy)-2,2-dimethylnaphtho[1,8-de]-[1,3]-dioxine (***32***)

2,2-Dimethylnaphtho [1,8-*de*]-[1,3]-dioxin-6-ol (**31**, 216 mg, 1 mmol), prepared from juglone (**1**) according to the literature [21], was dissolved in acetone (HPLC grade, 5 mL). Allyl bromide (430 μL, 601.14 mg, 5 equiv), followed by K_2_CO_3_ (207 mg, 1.5 equiv) were added to the solution, and the resulting mixture was gently stirred at rt for 18 h. The solid was then removed by filtration over a glass septum and then washed with 3 × 3 mL portions of DCM. Solvents were removed under vacuum, and the residue was purified by flash chromatography over silica gel (14 g). Elution with hexane/EtOAc afforded the well-known allyl ether **32** [22] (182 mg, yield = 71%) as a pale-yellow oil. (HRMS, ESI positive mode) C_16_H_17_O_3_^+^ [M + H]^+^ calc. 257,1172, found 257,1173. The IR (KBr) and ^1^H-NMR (300 MHz, CDCl_3_) spectra matched those reported in the literature [22]; ^13^C-NMR (75 MHz, CDCl_3_) δ_C_ 150.9, 149.5, 145.4, 133.3, 127.6, 126.7, 117.5, 117.0, 111.2, 111.1, 110.2, 106.4, 102.2, 69.7, 25.8.

### 2.15. 5-Allyl-2,2-dimethylnaphtho[1,8-de]-[1,3]-dioxin-6-ol (***33***)

Allyl ether **32** (350 mg, 1.37 mmol) was dissolved in dry degassed xylene (50 mL) under argon, and the resulting solution was refluxed vigorously for 6 h. Xylene was removed under vacuum, and the residue was purified by flash chromatography over silica gel (20 g). Elution with hexane/EtOAc, 90:10, afforded the well-known 2-allyl-naphtol **33** [22] (322 mg, yield = 92%) as a pale-yellow oil. HRMS (ESI positive mode) C_16_H_17_O_3_^+^ [M + H]^+^ calc. 257,1172, found 257,1173. The IR (KBr) and ^1^H-NMR (300 MHz, CDCl_3_) spectra matched those reported in the literature [22]. ^13^C-NMR (75 MHz, CDCl_3_) δ_C_ 150.0, 144.4, 144.3, 136.1, 126.8, 124.5, 124.3, 116.2, 116.0, 111.2, 111.1, 104.3, 102.0, 34.7, 25.8.

### 2.16. (E)-6-Methoxy-2,2-dimethyl-5-(prop-1-en-1-yl)naphtho[1,8-de]-[1,3]-dioxine (***34***)

MeI (390 μL, 889.2 mg, 8 equiv), followed by K_2_CO_3_ (162 mg, 1.5 equiv), was added to naphtol **33** (200 mg, 0.78 mmol) dissolved in dry acetone (HPLC Grade, 4 mL). The resulting mixture was gently stirred at 22 °C for 12 h. The solid was then removed by filtration over a glass septum and washed with DCM (3 × 3 mL). The solvents were removed under vacuum, and the resulting residue was purified by flash chromatography over silica gel (11 g). Elution with hexane/EtOAc, 99:1 afforded the *O*-methyl ether of naphthol **33** (200 mg, yield = 95%) as a pale-yellow oil, which was used immediately in the following step. HRMS (ESI positive mode) C_17_H_19_O_3_^+^ [M + H]^+^ calc. 271.1329, found 271.1330. ^1^H-NMR (300 MHz, CDCl_3_) δ_H_ 7.67 (1H, dd, *J* = 7.5, 1.5 Hz, 8-H), 7.30 (1H, t, *J* = 7.5 Hz, 7-H), 6.89 (1H, dd, *J* = 7.5, 1.5 Hz, 6-H), 6.56 (1H, s, 3-H), 5.95 (1H, tt, *J* = 13.5, 6.2 Hz, 2′-H), 5.00 (2H, ddt, *J* = 13.4, 2.1, 1.0 Hz, 3′-H_2_), 4.05 (3H, s, 1-OMe), 3.37 (2H, dt, *J* = 6.1, 1.0 Hz, 1′-H_2_), 1.65 (6H, s, 2 × Me). ^13^C-NMR (75 MHz, CDCl_3_) δ_C_ 150.9, 146.7, 146.4, 135.9, 127.4, 127.2, 126.79, 116.2, 115.6, 111.7, 111.1, 105.5, 102.0, 61.4, 34.1, 25.8. Pd(PhCN)_2_Cl_2_ (22 mg, 0.057 mmol, 0.045 equiv) was added to the freshly prepared methyl ether (342 mg, 1.27 mmol) dissolved in dry THF under Ar, and the resulting solution was refluxed for 52 h. Subsequently, the solvent was removed under reduced pressure to give an oily residue that was purified by flash chromatography over silica gel (15 g). Elution with hexane/EtOAc, 98:2, afforded olefin **34** (340 mg, yield = 99%) as a pale-yellow oil. HRMS (ESI positive mode) C_17_H_19_O_3_^+^ [M + H]^+^ calc. 271.1329, found. 271.1331. ^1^H-NMR (300 MHz, CDCl_3_) δ_H_ 7.67 (1H, dd, *J* = 7.5, 1.5 Hz, 8-H), 7.33 (1H, t, *J* = 7.5 Hz, 7-H), 7.14 (1H, s, 3-H), 6.89 (1H, dd, *J* = 7.5, 1.5 Hz, 6-H), 6.67 (1H, dq, *J* = 15.1, 1.0 Hz, 1′-H), 5.99 (1H, dq, *J* = 15.0, 6.4 Hz, 2′-H), 3.95 (3H, s, 1-OMe), 1.89 (3H, dd, *J* = 6.4, 1.0 Hz, 3′-H_3_), 1.65 (6H, s, 2 × Me). ^13^C-NMR (75 MHz, CDCl_3_) δ_C_ 151.0, 147.1, 145.9, 127.9, 127.9, 127.3, 126.6, 126.3, 115.9, 111.18, 107.0, 106.9, 102.0, 62.8, 25.8, 18.6.

### 2.17. 6-Methoxy-2,2-dimethylnaphtho[1,8-de]-[1,3]-dioxine-5-carbaldehyde (***35***)

NaIO_4_ (212 mg, 0.99 mmol, 2.15 equiv), followed by an aqueous solution of OsO_4_ (11.5 μL, 0.01 equiv), were added to propenylnaphthalene **34** (125 mg, 0.46 mmol) dissolved in THF/water (2:1, 4 mL). The resulting mixture was heated to 60 °C for 18 h, then cooled to 22 °C. Subsequently, an aqueous saturated solution of Na_2_S_2_O_3_ (5 mL) was added under vigorous stirring. The resulting mixture was transferred into a separating funnel and diluted with EtOAc (5 mL). The aqueous phase was separated and extracted with EtOAc (3 × 5 mL). The combined organic layers were dried over Na_2_SO_4_ and evaporated under vacuum to give an oily residue that was purified over silica gel (15 g). Elution with hexane/EtOAc, 9:1, afforded aldehyde **35** (96 mg, yield = 80%) as a pale-yellow oil. HRMS (ESI positive mode) C_15_H_15_O_4_^+^ [M + H]^+^ calc. 259.0970, found 259.0967. ^1^H-NMR (300 MHz, CDCl_3_) δ_H_ 10.45 (1H, s, C*H*O), 7.90 (1H, dd, *J* = 7.5, 1.5 Hz, 8-H), 7.50 (1H, t, *J* = 7.5 Hz, 7-H), 7.24 (1H, d, *J* = 0.5 Hz, 3-H), 6.94 (1H, dd, *J* = 7.5, 1.5 Hz, 6-H), 3.95 (3H, s, 1-OMe), 1.65 (6H, s, 2 × Me). ^13^C-NMR (75 MHz, CDCl_3_) δ_C_ 191.3, 150.3, 149.9, 147.7, 128.1, 127.9, 123.8, 118.3, 111.2, 109.7, 108.8, 102.0, 62.7, 25.8.

### 2.18. 1-(6-Methoxy-2,2-dimethylnaphtho[1,8-de]-[1,3]-dioxin-5-yl)but-3-en-1-ol (***36***)

Allylmagnesium bromide (1M in Et_2_O, 345 μL, 1.5 equiv) was added dropwise to a magnetically stirred solution of aldehyde **35** (60 mg, 0.23 mmol) in dry THF (3 mL) under Ar, cooled to 0 °C. Stirring continued for 1 h; then the reaction was quenched with saturated aqueous NH_4_Cl (5 mL,), and Et_2_O (10 mL) was added to the mixture. The organic layer was separated, and the aqueous phase was extracted with more Et_2_O (3 × 5 mL). The combined organic layers were dried over Na_2_SO_4_ and evaporated under vacuum to give an oily residue that was separated over silica gel (12 g). Elution with hexane/EtOAc, 9:1, gave homoallyl alcohol **36** (63 mg, yield = 91%) as a pale-yellow oil. HRMS (ESI positive mode) C_18_H_21_O_4_^+^ [M + H]^+^ calc. 301.1434, found 301.1433.^1^H-NMR (300 MHz, CDCl_3_) δ_H_ 7.72 (1H, dd, *J* = 7.8, 1.2 Hz, 8-H), 7.12 (1H, dd, *J* = 7.9, 7.1 Hz, 7-H), 6.98–6.85 (2H, m, 3-H and 8-H), 5.89–5.68 (1H, m, 3′-H), 5.22–5.05 (2H, m, 4′-H_2_), 5.01–4.87 (1H, m, 1′-H), 3.87 (3H, s, 1-OMe), 3.50 (1H, s, O*H*), 2.61 (2H, dtd, *J* = 14.7, 6.9, 1.4 Hz, 2′-H_2_), 1.74–1.64 (6H, 2x s, 2 × Me). ^13^C-NMR (75 MHz, CDCl_3_) δ_C_ 148.8, 146.5, 144.5, 134.7, 133.4, 128.6, 127.5, 118.0, 114.6, 113.4, 109.5, 106.1, 101.7, 68.1, 62.7, 42.9, 26.3, 24.8.

### 2.19. 1-(6-Methoxy-2,2-dimethylnaphtho[1,8-de]-[1,3]-dioxin-5-yl)-4-methylpent-3-en-1-ol (***38***)

Alcohol **38**, pale yellow oil, was prepared in 80% yield from compound **36** with the metathesis reaction described in Section 2.3.2. HRMS (ESI positive mode) C_20_H_25_O_4_^+^ [M + H]^+^ calc. 329.1753, found 329.1748; ^1^H-NMR (300 MHz, CDCl_3_) δ_H_ 7.72 (1H, dd, *J* = 7.8, 1.2 Hz, 8-H), 7.12 (1H, dd, *J* = 7.9, 7.1 Hz, 7-H), 6.98–6.85 (2H, m, 3- and 6-H), 5.28–5.14 (1H, m, 3′-H), 4.99 (1H, tddd, *J* = 6.9, 6.1, 1.8, 0.7 Hz, 1′-H), 3.87 (3H, s, 1-OMe), 2.78–2.60 (1H, m, 2′-H_A_), 2.52–2.35 (1H, m, 2′-H_B_), 1.68–1.57 (12H, m, acetal-Me_2_ and 4′-Me_2_); ^13^C-NMR (75 MHz, CDCl_3_) δ_C_ 150.8, 149.4, 147.5, 134.9, 133.4, 127.6, 126.5, 120.1, 115.6, 111.4, 110.5, 110.1, 102.9, 67.7, 61.6, 35.8, 26.3, 25.6, 20.0.

### 2.20. 1-(6-Methoxy-2,2-dimethylnaphtho[1,8-de]-[1,3]-dioxin-5-yl)-4-methylpent-3-en-1-yl Acetate (***39***)

Pyridine (18.7 mg, 0.24 mmol), followed by Ac_2_O (24.5 mg, 0.24 mmol) and a catalytic amount of DMAP, was added to a solution of alcohol **38** (40 mg, 0.12 mmol) dissolved in DCM (4 mL), cooled to 0 °C. The resulting mixture was stirred overnight at 22 °C; then the reaction was quenched by adding MeOH (50 μL), followed by saturated aqueous NaHCO_3_ (5 mL) and DCM (6 mL). The organic layer was separated, and the aqueous phase was extracted with DCM (3 × 5 mL). The combined organic layers were washed with brine (3 mL), dried over Na_2_SO_4_, and evaporated. The resulting residue was separated on a silica gel column (10 g). Elution with hexane/EtOAc, 9:1, gave acetate **39** as a pale-yellow oil (42 mg, yield = 95%). HRMS (ESI positive mode) C_22_H_27_O_5_^+^ [M + H]^+^ calc. 371.1858, found 371.1854.^1^H- NMR (300 MHz, CDCl_3_) δ_H_ 7.72 (1H, dd, *J* = 7.8, 1.2 Hz, 8-H), 7.12 (1H, dd, *J* = 7.9, 7.1 Hz, 7-H), 6.98 (1H, d, *J* = 0.6 Hz, 3-H), 6.90 (1H, dd, *J* = 7.0, 1.2 Hz, 6-H), 6.04 (1H, tdd, *J* = 6.5, 1.8, 0.7 Hz, 1′-H), 5.36–5.22 (1H, m, 3′-H), 3.87 (3H, s, OMe), 2.90–2.73 (1H, m, 2′-H_A_), 2.65–2.47 (1H, m, 2′-H_B_), 2.08 (3H, s, *Me*CO), 1.68–1.57 (12H, m, acetal-Me_2_ and 4′-Me_2_). ^13^C-NMR (75 MHz, CDCl_3_) δ_C_ 169.9, 150.6, 148.3, 147.2, 135.9, 130.6, 127.6, 125.9, 118.4, 115.8, 111.4, 110.8, 110.1, 102.9, 72.4, 61.6, 33.1, 26.3, 25.5, 21.0, 19.8.

### 2.21. ((5-Allyl-2,2-dimethylnaphtho[1,8-de]-[1,3]-dioxin-6-yl)oxy)(tert-butyl)dimethylsilane (***40***)

Imidazole (Im) (113 mg, 1.66 mmol, 2 equiv), followed by TBSCl (137.5 mg, 0.91 mmol, 1.1 equiv), was added to naphtol **33** (213 mg, 0.83 mmol) dissolved in dry DCM (8.3 mL). The resulting mixture was stirred at 22 °C for 24 h. Subsequently, the organic layer was washed with a saturated aqueous solution of NaHCO_3_ (5 mL), and the aqueous phase was extracted with DCM (3 × 4 mL). The combined organic layers were dried over Na_2_SO_4_ and evaporated under reduced pressure. The resulting oily residue was purified by flash chromatography over a silica gel column (15 g). Elution with hexane/EtOAc, 98:2, gave compound **40** (340 mg, yield = 99%) as a pale-yellow oil. HRMS (ESI positive mode) C_22_H_31_O_3_Si^+^ [M + H]^+^ calc. 371.2042, found 371.2038. ^1^H-NMR (300 MHz, CDCl_3_) δ_H_ 7.65 (1H, d, *J* = 8.2 Hz, 8-H), 7.40 (1H, dd, *J* = 8 and 7.5 Hz, 7-H), 6.83 (1H, d, *J* = 7.5 Hz, 6-H), 6.4 (1H, s, 3-H), 6.15–5.95 (1H, ddt, *J* = 16.7, 10.5 and 6.1 Hz, 2′-H), 5.31 (1H, d, *J* = 10.5 Hz, 3′-H*_Z_*), 5.20 (1H, d, *J* = 16.7 Hz, 3′-H*_E_*), 3.55 (2H, d, *J* = 6.1 Hz, 1′-H_2_), 1.65 (6H, s, Me_2_), 0.9 (9H, s, *tert*-BuSi), 0.15 (6H, s, Me_2_Si). ^13^C-NMR (75 MHz, CDCl_3_) δ_C_ 150.7, 148.0, 139.7, 136.1, 128.3, 127.3, 126.7, 116.2, 115.8, 111.8, 111.5, 108.5, 102.9, 33.9, 26.32, 25.6, 18.3, −4.3.

### 2.22. Tert-butyl((2,2-dimethyl-5-(3-methylbut-2-en-1-yl)naphtho[1,8-de]-[1,3]-dioxin-6-yl)oxy)dimethylsilane (***41***)

Compound **40** was converted in 75% yield into pale-yellow oily olefin **41** with the metathesis reaction described in Section 2.3.2. HRMS (ESI positive mode) C_24_H_35_O_3_Si^+^ [M + H]^+^ calc. 399.2355, found 399.2358; ^1^H-NMR (300 MHz, CDCl_3_) δ_H_ 7.68 (1H, dd, *J* = 7.8, 1.2 Hz, 8-H), 7.18 (1H, dd, *J* = 7.9, 7.1 Hz, 7-H), 6.93 (1H, dd, *J* = 7.0, 1.2 Hz, 6-H), 6.63 (1H, t, *J* = 0.8 Hz, 3-H), 5.31 (1H, tdd, *J* = 7.4, 1.8, 1.3 Hz, 2′-H), 3.34 (2H, ddq, *J* = 7.5, 1.9, 1.0 Hz, 1′-H_2_), 1.73 (6H, br s, 3′-Me_2_), 1.64 (6H, s, Me_2_), 1.01 (9H, s, *tert*-BuSi), 0.24 (6H, s, Me_2_Si); ^13^C-NMR (75 MHz, CDCl_3_) δ 151.1, 148.5, 140.1, 133.3, 128.9, 127.7, 126.7, 122.3, 115.8, 111.8, 111.5, 108.5, 102.9, 28.4, 26.3, 25.7, 25.6, 19.8, 18.2, −4.3.

### 2.23. 2-(6-((Tert-butyldimethylsilyl)oxy)-2,2-dimethylnaphtho[1,8-de]-[1,3]-dioxin-5-yl)ethan-1-ol (***42***)

9-BBN (0.4 M in hexane, 0.95 mL, 0.38 mmol, 2 equiv) was added dropwise to a solution of *O*-TBS ether **40** (70 mg, 0.19 mmol) in dry THF (2 mL) under Ar, cooled to 0 °C. Subsequently, the ice bath was removed, and the reaction mixture was stirred at 22 °C for 12 h until starting material, visualized on a TLC plate, disappeared. Subsequently, the reaction mixture was cooled to 0 °C, and NaOH (1M, 0.38 mL, 2 equiv), followed by excess 30% aqueous H_2_O_2_, was added under vigorous stirring. The resulting mixture was stirred at 22 °C for 4h, and then it was diluted with Et_2_O (10 mL) and water (5 mL). The organic layer was separated, and the aqueous phase was extracted with Et_2_O (4 × 8 mL). The combined organic layers were washed with H_2_O (10 mL), dried over Na_2_SO_4_, and concentrated under reduced pressure to give an oily residue that was separated by flash chromatography over a silica gel column (15 g). Elution with hexane/EtOAc, 98:2, afforded alcohol **42** (70 mg, yield = 95%) as a pale-yellow oil. HRMS (ESI positive mode) C_22_H_33_O_4_Si^+^ [M + H]^+^ calc. 389.2148, found 389.2144. ^1^H-NMR (300 MHz, CDCl_3_) δ_H_ 7.65 (1H, d, *J* = 8.3, Hz, 8-H), 7.41 (1H, dd, *J* = 8.2 and 7.5 Hz, 7-H), 6.85 (1H, d, *J* = 7.5 Hz, 6-H), 6.4 (1H, s, 3-H), 3.5 (2H, m, 3′-H_2_), 2.95–2.85 (2H, m, 1′-H_2_), 1.98–1.93 (2H, m, 2′-H_2_), 1.65 (6H, s, Me_2_), 0.9 (9H, s, *tert*-Bu), 0.12 (6H, s, *Me*_2_Si). ^13^C-NMR (75 MHz, CDCl_3_) δ_C_ 150.6, 148.1, 140.2, 129.5, 127.3, 126.5, 115.8, 112.0, 111.5, 108.5, 102.9, 62.1, 31.5, 28.0, 26.3, 25.6, 18.3, −4.3.

### 2.24. Tert-butyl((2,2-dimethyl-5-(4-methylpent-3-en-1-yl)naphtho[1,8-de]-[1,3]-dioxin-6-yl)oxy)dimethylsilane (***43***)

Dess-Martin periodinane (DMP, 165 mg, 1.2 equiv) was added to a solution of alcohol **42** (110 mg, 0.283 mmol) in dry DCM under Ar, and the resulting mixture was stirred at 22 °C for 2h. The reaction was then quenched with saturated aqueous Na_2_S_2_O_3_ (5 mL) and NaHCO_3_ (3mL) and diluted with DCM (7 mL). The organic layer was separated, and the aqueous phase was extracted with DCM (3 × 10 mL). The combined organic layers were washed with H_2_O (8 mL), dried over Na_2_SO_4_, filtered, and concentrated under reduced pressure to give a crude aldehyde (^1^H-NMR signal of C*H*O at δ_H_ 9.82) which was directly used in the subsequent olefination step. Isopropyl triphenyl phosphonium bromide (330 mg, 0.86 mmol, 3 equiv) was suspended in dry THF (5 mL) contained in a two-neck round-bottom flask under an argon atmosphere. The resulting suspension was cooled to 0 °C, and potassium bis(trimethylsilyl)amide (KHMDS, 0.5 M in toluene, 1.72 mL, 3 equiv) was added dropwise. The deep red solution of the formed ylide was stirred at 22 °C for 45 min; subsequently, a solution of the crude aldehyde in dry THF (2 mL) was added dropwise, and the mixture was stirred for an additional 3 h at 22 °C. Once completed, the reaction was quenched with saturated aqueous NH_4_Cl, and the mixture was diluted with Et_2_O (15 mL). The organic layer was separated, and the aqueous phase was extracted with Et_2_O (3 × 10 mL). The combined organic layers were washed with H_2_O (8 mL), dried over Na_2_SO_4_, filtered, and concentrated under reduced pressure to give a residue which was separated by flash chromatography over a silica gel column (15 g). Elution with hexane/EtOAc, 99:1, afforded compound **43** (105 mg, yield = 90% from **42**) as a pale-yellow oil. HRMS (ESI positive mode) C_25_H_37_O_3_Si^+^ [M + H]^+^ calc. 413.2512, found 413.2507. ^1^H-NMR (300 MHz, CDCl_3_) δ*_H_* 7.68 (1H, d, *J* = 8.5 Hz, 8-H), 7.26 (1H, dd, *J* = 8.5 and 7.5 Hz, 7-H), 6.79 (1H, d, *J* = 7.5 Hz, 6-H), 6.72 (1H, s, 3-H), 5.18 (1H, br t, *J* = 7.2 Hz, 3′-H), 2.75 (2H, dd, *J* = 7.2 and 7.5 Hz, 1′-H_2_), 2.29 (2H, br q, *J* = 7.3 Hz, 2′-H_2_), 1.72 (3H, br s, 4′-Me*_E_*), 1.67 (6H, s, 2 × Me), 1.57 (3H, br s, 4′-Me*_Z_*), 1.09 (9H, s, *tert*-Bu), 0.18 (6H, s, *Me*_2_Si). ^13^C-NMR (75 MHz, CDCl_3_) δ_C_ 150.6, 147.9, 140.5, 132.8, 128.8, 127.3, 126.5, 123.1, 115.8, 112.0, 111.5, 108.5, 102.9, 30.3, 27.4, 26.3, 25.6, 25.5, 19.6, 18.3, −4.4.

### 2.25. 1-(6-Methoxy-2,2-dimethylnaphtho[1,8-de]-[1,3]-dioxin-5-yl)propan-1-ol (***44***)

Freshly purified *m*-chloroperoxybenzoic acid (*m*-CPBA, 51.7 mg, 0.15 mmol, 1.5 equiv) and powdered NaHCO_3_ (12.6 mg, 0.15 mmol, 1.5 equiv.) were added in a single portion to a solution of olefin **34** (27 mg, 0.1 mmol,) in dry DCM (2 mL) at 0 °C under Ar. The resulting mixture was stirred at 0 °C for 8 h; subsequently, the reaction was quenched by adding saturated aqueous NaHCO_3_ (4 mL) and DCM (16 mL). The organic layer was separated, and the aqueous phase was extracted with DCM (3 × 15 mL). The combined organic layers were washed with H_2_O (8 mL), dried over MgSO_4_, filtered, and evaporated under vacuum to give an oily residue (28.4 mg, quantitative yield) which was used directly in the next step, as the formed epoxide degraded rapidly on silica gel. Thus, LiAlH_4_ (1M in Et_2_O 0.37 mL, 4.0 equiv) was added to a solution of crude epoxide (26.5 mg, 0.092 mmol) in dry THF (4 mL) which was magnetically stirred for 1h at −30 °C, under Ar. Subsequently, the temperature was allowed to rise to 22 °C, and once completed, the reaction was quenched by adding saturated aqueous potassium-sodium tartrate (4 mL), H_2_O (8 mL) and Et_2_O (4 mL). The resulting mixture was stirred at 22 °C until two layers were clearly observed. The layers were separated, and the aqueous phase was extracted with DCM (3 × 8 mL). The combined organic layers were dried over Na_2_SO_4_, filtered, and concentrated under vacuum. The resulting residue was chromatographed on a silica gel column (10 g). Elution with hexane-EtOAc, 90:10, afforded benzylic alcohol **44** (13.9 mg, yield = 52%) as a yellowish oil. HRMS (ESI positive mode) C_17_H_21_O_4_^+^ [M + H]^+^ calc. 289.1440, found 289.1434. ^1^H NMR (300 MHz, CDCl_3_) δ_H_ 7.72 (1H, dd, *J* = 7.8, 1.2 Hz, 8-H), 7.12 (1H, dd, *J* = 7.9, 7.1 Hz, 7-H), 6.97–6.85 (2H, m, 6- and 3-H), 4.93–4.80 (1H, m, 1′-H), 3.87 (3H, s, 1-OMe), 3.38–3.30 (1H, m, 2′-H_a_), 1.88 (1H, dqd, *J* = 13.1, 7.5, 5.5 Hz, 2′-H_b_), 1.64 (6H, s, 2 × Me), 1.04 (3H, td, *J* = 7.5, 1.5 Hz, 3′-H_3_). ^13^C NMR (75 MHz, CDCl_3_) δ_C_ 150.7, 149.7, 147.3, 136.2, 126.3, 124.4, 113.3, 111.1, 110.9, 108.7, 103.7, 74.5, 63.3, 31.3, 26.3, 14.8.

### 2.26. MTT Test

The inhibitory activities, expressed as IC_50_ (μM/L) indices, of compounds **1**, **3**, **15**, **16**, **17**, (±)-**18** and the corresponding enantiomers **18a** and **18b**, **19**, **20**, and **21**, on the viability of non-small lung cancer H460 cells and breast cancer MCF-7 cells, were determined spectrophotometrically with the colorimetric MTT [3-(4,5-dimethylthiazol-2-yl)-2,5-diphenyltetrazolium bromide] assay [19]. The test was based on the conversion of pale-yellow MTT to deep purple MTT-formazan by mitochondrial enzymes of viable cells: thus, the darker the solution, the greater the number of viable, metabolically active cells.

#### 2.26.1. Cell Cultures

H460 and MCF-7 cells were purchased from ATCC (Manassas, VA, USA) and were cultured in the dark in RPMI-1640 medium (Hyclone; Thermo Fisher Scientific, Rockford, IL, USA) containing 10% FBS (phosphate-buffered saline), 100 U/mL penicillin and 100 µg/mL streptomycin at 37 °C under a humid atmosphere of 95% air and 5% CO_2_.

#### 2.26.2. Cell Viability Test

Cells were harvested during the exponential phase of growth, seeded in quadruplicate at about 5 × 10^3^ cells/well in 96-well plates, and incubated overnight. Each tested sample (100 mL/well) was prepared in dimethyl sulfoxide (DMSO) and subsequently diluted with the RPMI-1640 medium prior to use in a range of serial concentrations from 0.1 to 50 µM). The final concentration of DMSO was less than 0.1%. The control group received the same amount of DMSO. Cells were exposed to each tested sample and then incubated for 48 h. Subsequently, the medium was removed, and the cells were washed with PBS; subsequently, 10 mL of a stock MTT solution (5 mg/mL in PBS; Sigma–Aldrich, Milano (MI), Italy) was added to each well, and the cells were incubated for an additional 4 h at 37 °C until intracellular purple formazan was visible under a microscope. Subsequently, the culture medium containing unreacted dye was removed, and the cells were exposed to DMSO (150 µL) and incubated at 37 °C until they lysed and formed purple formazan was completely dissolved. The absorbance (*Abs*) of the resulting solution was measured at 570 nm using an automated microplate reader (BioTek, Hercules, CA, USA). Each assay was repeated in three separate experiments, and mean absorbance ± SD (standard deviation) was calculated. The percentage cell viability and the percentage inhibition of cell viability were calculated by the equations [(*Abs*_sample_ − *Abs*_blank_)/*Abs*_control_ − *Abs*_blank_)] × 100 and [(*Abs*_control_ − *Abs*_blank_ − *Abs*_sample_)/*Abs*_control_ − *Abs*_blank_] × 100, respectively, where the control was untreated cells, and the blank was the medium. A sample was considered non-cytotoxic if the cell viability was >70% for all concentrations; instead, if the cell viability was <70% for at least one concentration, the half-maximal inhibitory concentration (IC_50_, μM/L) index was calculated by probit analysis (*p* < 0.05, χ^2^ test), plotting the percentage cell viability versus sample concentration. An IC_50_ index denotes the concentration of a compound at which 50% of cell viability is inhibited or, as a complement, 50% of cells survive. Commercially available juglone (**1**) and shikonin (**3**) were the reference compounds.

### 2.27. Mulliken Population Analysis of the Regioisomeric Anions Derived from the Deprotonation of Compound ***25***

Calculations were carried out using the Gaussian09D program [23] at a differentiated level of theory [6-31+G(d,p)] with the DFT/B3LY functional [24,25]. The role of the solvent THF was simulated by using the polarizable continuum model (PCM) theory [26]. The computed Cartesian coordinates of compound **25** and the charge distributions in the regioisomeric species resulting from the deprotonation of compound ***25*** are reported in Table S1 and Figures S10–S12, respectively, in the Supplementary Materials.

## 3. Results and Discussion

### 3.1. Synthetic Routes to 2- and 3-Substituted Juglone Derivatives

**Scheme 1:**
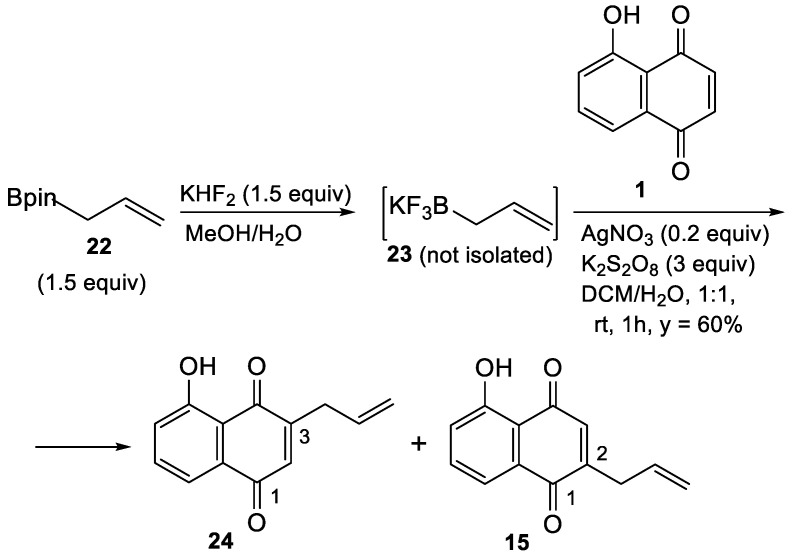
Direct allylation of juglone (**1**) with the Baran method.

Synthetic strategies directed to the prenylation of aromatics include the Friedel–Crafts reaction, C–H activation, the Tsuji–Trost reaction, addition reactions, cross-coupling and radical coupling [27]. [In particular, the allylation/prenylation of quinones includes the radical prenylation of symmetrical 1,4-naphthoquinone with 4-methyl-3-pentenoic acid in the presence of silver nitrate and ammonium persulfate, the Pd-catalyzed Stille cross-coupling of a prenylstannane with a prehalogenated quinone [28], and the allylation of quinones by allylic indium sesquihalides [29]. However, the direct prenylation of quinones with prenyl bromide in MeCN, using a catalytic amount of lead bromide and aluminum powder [30], appeared to be a more attractive method. In fact, this procedure was also applied to the mono-prenylation of juglone, although the structure of the product was not specified [30]. In our hand, however, the reaction was not reproducible, and therefore the method was abandoned. Subsequently, the direct C-H functionalization of juglone with an allylboronic acid was explored, according toa the procedure developed by Baran and his group for the coupling of alkyl- and arylboronic acids to 1,4-benzoquinones [31]. Thus, pinacolboronate **22** was first converted into the corresponding Molander salt **23** (not isolated), which was subjected to the standard Baran conditions [31] to bind it directly to the juglone nucleus (Scheme 1). In the event, the reaction was not regioselective, leading in 60% yield to a chromatographically inseparable 1.4:1 mixture (quantified by ^1^H NMR) of 3- (**24**) and 2-allyl juglone (**15**) which was identical with a sample prepared by an alternative route (see Section 3.3 below).

**Scheme 2:**
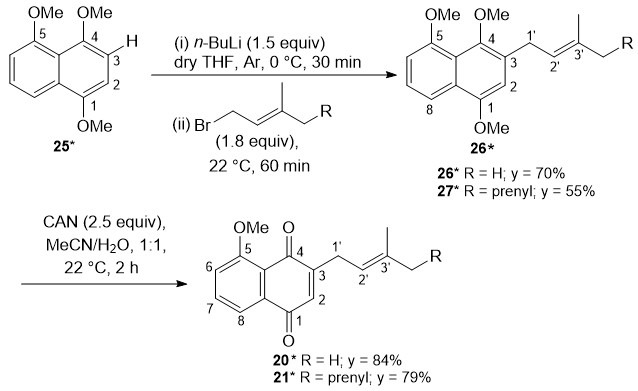
Regioselective prenylation of non-symmetric 1,4,5-trimethoxynaphthalene (**25**). * The compound was numbered using the same numbering system as juglone (**1**).

After these unsatisfactory experiments, we envisaged that the regioselective synthesis of a prenylated juglone derivative could be carried out by *ortho*-metalation of the corresponding hydroquinone methyl ether [32], followed by addition of a prenyl halide and oxidative demethylation with ceric (IV) ammonium nitrate (CAN) [33,34,35]. However, most *ortho*-metalation reactions reported in the literature involved symmetric phenolic derivatives or derivatives that have already been desymmetrized and contained two or fewer oxygen substituents; in contrast, the reactions with non-symmetric phenol methyl ethers have been less investigated [32]. In this context, we explored the regiochemistry of the prenylation of commercially available 1,4,5-trimethoxynaphthalene (**25**) as a possible entry to the corresponding prenyl juglone derivative (Scheme 2). In principle, the *ortho*-deprotonation of 1,4,5-trimethoxynaphthalene (**25**) with *n*-BuLi could afford three different negatively charged species, A, B and C, at C(2), C(3) or C(6), respectively (Figures S10–S12 in the Supplementary Materials); therefore, three different regioisomeric products could result from the subsequent monoprenylation reaction. However, the Mulliken population analysis [36] of the three species A-C revealed that the negative charge densities at the positions *ortho* to the methoxy groups were significantly different (Figures S10–S12 in the Supplementary Materials), being −2.978 at C(3), −2.224 at C(2) and −1.320 at C(6). Thus, we envisioned that the directed *ortho*-deprotonation of naphthalene **25**, followed by prenylation and CAN oxidative demethylation, would have mainly afforded a 3-substituted juglone derivative.

### 3.2. Regioselective Prenylation of Non-Symmetric Naphthoquinone ***25***

As anticipated, treatment of compound **25** with BuLi (2.5 M, 1.5 equiv) in THF at 0 °C for 30 min, followed by addition of prenyl bromide (1.8 equiv), yielded 3-prenyl naphthalene **26** in good yield (70%) (Scheme 2), accompanied by two unidentified regioisomers. The structure of product **26** was established from the ^3^J-HMBC correlation between 2-H resonating at δ_H_ 6.65 and C-1′ (δ_C_ 28.8); moreover, selected NOE irradiation of 1-OMe at δ_H_ 3.95 enhanced the signals of 2-H (14%) at δ_H_ 6.65 and 8-H (2%) at δ_H_ 7.81, while irradiation of 5-OMe at δ_H_ 3.99 enhanced the signal of 6-H (16%) at δ_H_ 6.88, and irradiation of 1′-H_2_ at δ_H_ 3.53 enhanced the signals of 2-H (3,5%) at δ_H_ 6.65, 4-OMe (1%) at δ_H_ 3.84, 2′-H (4%) at δ_H_ 5.35 and 3′-Me*_Z_* (1.5%) at δ_H_ 1.74. Subsequent CAN oxidation of 1,4-dimethoxynaphthalene **26** in aqueous MeCN at 22 °C [30,31,32,33,34,35] afforded naphthoquinone **20** in 84% yield (Scheme 2). Interestingly, structure **20** corresponded to one of the two possible structures assigned to a natural naphtoquinone isolated from the roots of *Rubia cordifolia* [37].

Preferential prenylation of deprotonated 1,4,5-trimethoxynaphthalene (**25**) also took place at C(3) using geranyl bromide as an electrophile (Scheme 2). The product structure, **27**, was firmly established by selected NOE experiments. Thus, the irradiation of 1-OMe at δ_H_ 3.94 enhanced the signals of 2-H (16%) at δ_H_ 6.67 and 8-H (2%) at δ_H_ 7.83, while the irradiation of 5-OMe at δ_H_ 4.00 enhanced the signal of 6-H (17%) at δ_H_ 6.89, and the irradiation of 1′-H_2_ at δ_H_ 3.56 enhanced the signals of 2-H (4%) at δ_H_ 6.67, 4-OMe (1%) at δ_H_ 3.77, 2′-H (3%) at δ_H_ 5.35, and 3′-Me (2%) at δ_H_ 1.79. Subsequent CAN oxidation of compound **27** yielded 3-geranyl-5-*O*-methyl juglone (**21**) in 79% yield (Scheme 2).

**Scheme 3:**
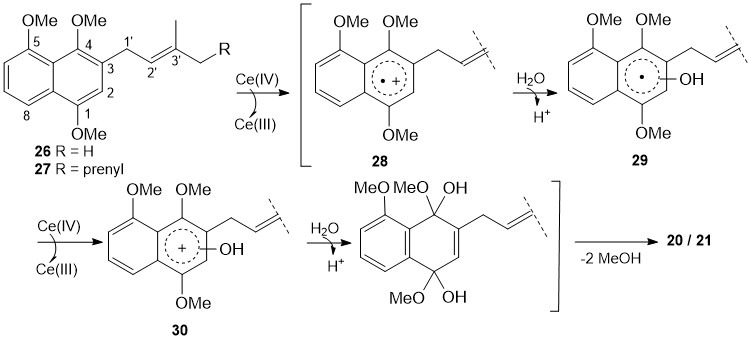
The proposed mechanism for the CAN-mediated oxidative demethylation of compounds **26** and **27** to **20** and **21**, respectively.

Of note, in the CAN oxidative demethylation of compounds **26** and **27**, the oxidation of allylic methylene was not observed (Scheme 2). The regioselective CAN oxidation of prenyl dihydroquinone methyl ethers is well-known [33,34] and, in the case of **26** and **27**, could be ascribed to the presence of the electron-donating methoxy groups on C(1) and C(4)*,* which greatly enriched the electron density of the aromatic ring. This electronic effect resulted in an increase in the chemical reactivity of the aromatic ring, compared to the allylic C(1′) position, thus facilitating the transfer of a single electron from **26** or **27** to Ce(IV) to give the resonance-stabilized aryl radical **28** (Scheme 3). Moreover, according to the mechanism proposed for the reaction [33,38], the two methoxy groups also contributed to the stabilization of the cation ion **30** formed by another single-electron transfer from intermediate **29** to Ce(IV) (Scheme 3).

### 3.3. Regioselective Synthesis of 2-Allyl-Juglone (***15***)

**Scheme 4:**
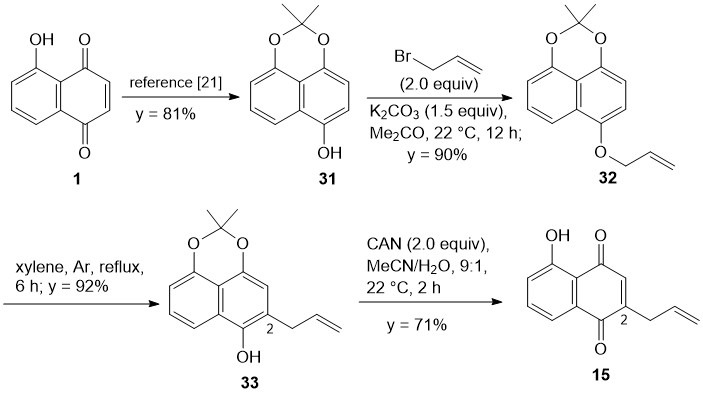
Regioselective synthesis of 2-allyl-juglone (**15)**.

Alternatively to the *ortho*-metalation of trimethoxynaphthalene **25**, the well-known Claisen rearrangement of allyl ether **32** [22] was envisioned as a straightforward regioselective entry to 2-substituted juglones (Scheme 4). The well-known 2,2-dimethylnaphtho [1,8-de]-[1,3]-dioxin-6-ol (**31**) was prepared in two steps (yield = 81%) from juglone (**1**) according to a published procedure [21]. Subsequently, etherification of naphthol **31** with allyl bromide in Me_2_CO at 22 °C, according to a slightly modified literature protocol [22], gave allyl ether **32**, whose IR and ^1^H-NMR spectra were identical to those in the literature [22]. Then, by modifying the method described in the literature [22], thermal rearrangement of **32** in xylene at reflux for 6 h readily delivered the well-known 2-allyl naphthalene derivative **33** [22], in excellent yield (92%). Finally, regioselective CAN oxidation of 2-allyl naphthol **33** in wet MeCN afforded 2-allyl juglone **15** in 71% yield (Scheme 4). This compound was identical to the minor regioisomer obtained by direct allylation of juglone (**1**) with the Baran method (Scheme 1 above).

### 3.4. Synthesis of 2-(1-Acetoxy-4-methylpent-3-en-2-yl)juglone (***18***)

**Scheme 5:**
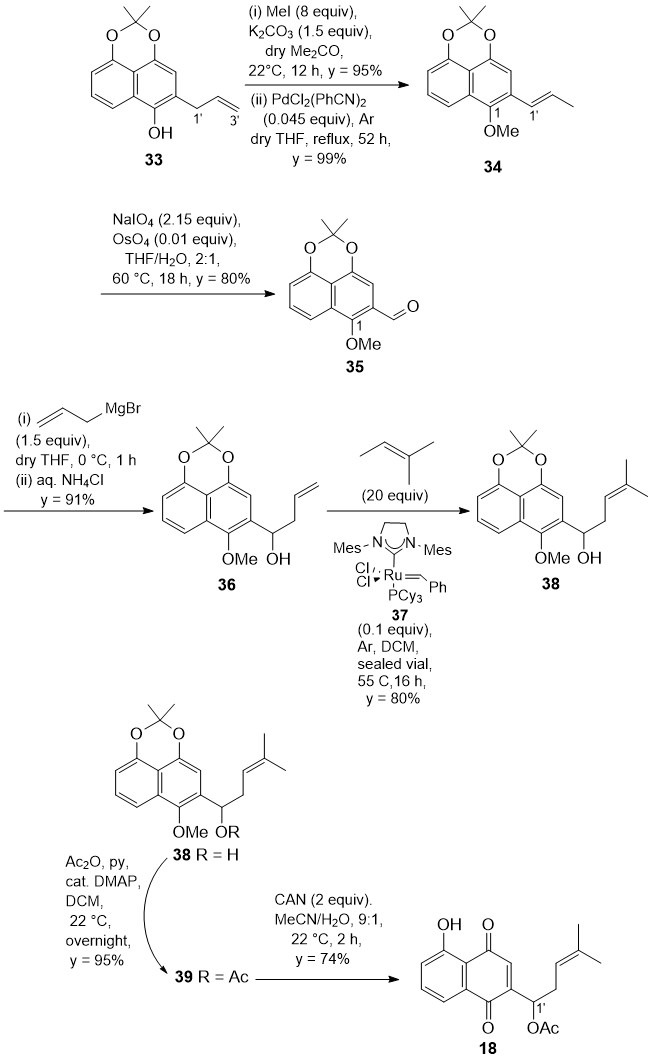
Regioselective synthesis of the juglone derivative **18**.

2-Allyl naphthol (**33**) was also used as the starting material in a straightforward synthesis (Scheme 5) of our main synthetic goal, compound (±)-**18**, which was characterized by the same sidechain at C(2) as the reference shikonin and alkannin acetates (**4** and **8**, Figure 1).

Initially, compound **33** was protected as 1-*O*-methyl ether to avoid the cycloetherification reaction of the free phenol onto the adjacent olefin in the Pd(II)-catalyzed isomerization of the allylic double bond to styrene olefin **34**. The structure of compound **34** was firmly confirmed by the NOE interaction between 1-OMe at δ_H_ 3.95 and 1′-H at δ_H_ 6.67. Subsequently, the styrene double bond of **34** was oxidatively cleaved to afford aldehyde **35** in 75% overall yield from phenol **33**. Addition of allyl magnesium bromide to aldehyde **35** readily afforded homoallylic alcohol **36** which was then treated with excess 2-methyl-2-butene and the 2nd generation Grubbs catalyst **37** [20]. The metathesis reaction proceeded smoothly, affording homoallylic alcohol **38** in 80% yield. The synthesis of 2-(1-acetoxyhomoprenyl) juglone **18** was then completed uneventfully by standard acetylation of alcohol **38**, followed by CAN oxidative demethylation [33,34,35] of the resulting acetate **39**. 2-Substituted juglone (±)-**18** was thus obtained with a remarkable yield of 51.2% over four steps from aldehyde **35**. Spectroscopic data fully supported the structures of all the compounds prepared in the synthesis from **35** to **18**.

### 3.5. Synthesis of Other Representative 2-Substituted Juglone Derivatives

**Scheme 6:**
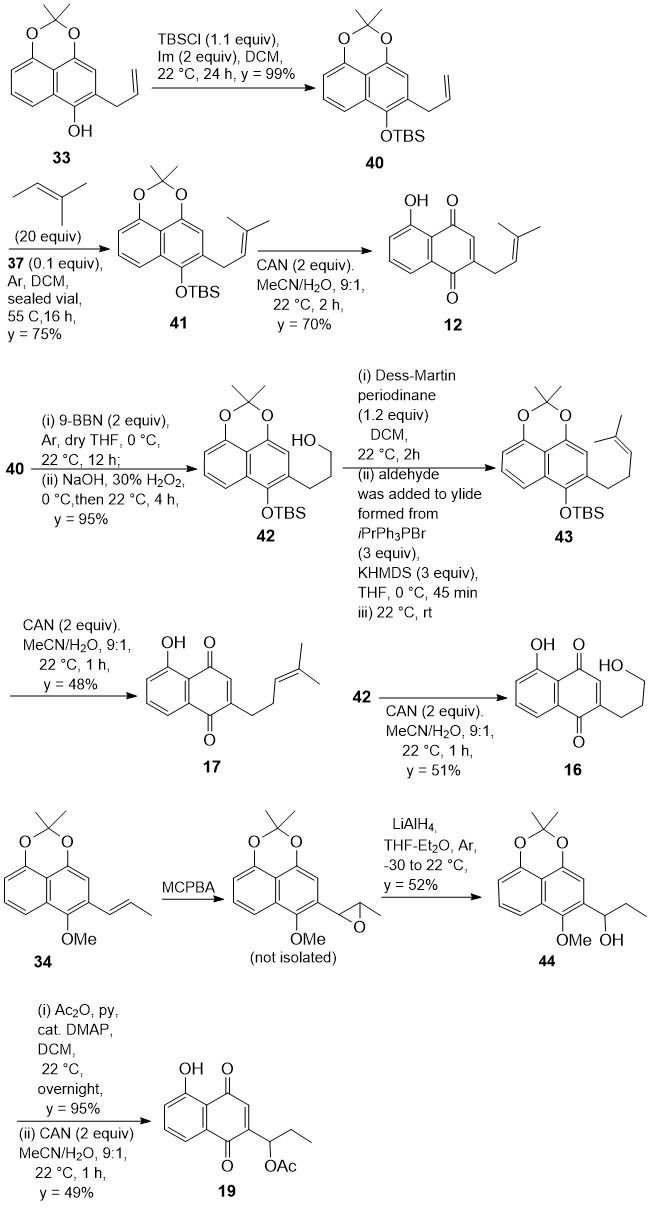
Synthesis of juglone derivatives **12**, **16**, **17**, and **19**.

To widen the spectrum of synthetic 2-substituted juglone derivatives, the intermediates **33** and **34** were also readily converted into compounds **12**, **16**, **17**, and **19**. Standard reagents and reaction conditions were used (Scheme 6) that do not require comments. The structures of synthetic intermediates as well as final products were fully supported by spectroscopic data.

### 3.6. Inhibition of the Viability of Human Cancer H460 and MCF-7 Cells (MTT Test)

To gain a preliminary insight into the relationship between structure and inhibitory activity of tumor cell viability, compounds **15**, **16**, **17**, (±)-**18**, **19**, **20**, and **21** were tested in vitro with the MTT test [19] against human cancer H460 and MCF-7 cells. Enantiomers **18a**–**b**, obtained from the HPLC resolution of racemic **18** on a semi-preparative ChiralPack AS-H column (Paragraph 2.8), were also included in the test, although the paucity of isolated material prevented the determination of the absolute configuration of each enantiomer. Commercially available juglone (**1**) and shikonin (**3**) were used as reference compounds. H460 cells are human-derived large cell lung cancer cells involved in cell mutations, proliferation, growth, invasion, and metastasis. These cells are thus commonly used in lung carcinoma and toxicology research and are a valuable tool for studying various cancer biology aspects involving tumor development, growth, and drug resistance. On the other hand, MCF-7 has been a commonly used breast cancer cell line by multiple groups since it has been proven to be a suitable model for breast cancer investigations worldwide, especially for studying estrogen receptor-positive breast cancers [39]. In conclusion, both cell lines are appropriate models for discovering and developing anti-cancer drugs. Table 1 shows the IC_50_ (μM/L) indices determined with the MTT test.

IC_50_ (Table 1) clearly indicated that the viability of H460 and MCF-7 cancer cells was significantly affected by the introduction of a substituent at the C(2) or C(3) position of the juglone nucleus (**1**), although the inhibitory potency of all tested juglone derivatives was lower than that of shikonin (**3**). In general, H460 cells were more sensitive to tested compounds than MCF-7 cells. Juglone derivatives substituted by a side chain longer than three carbons and ending with an OH group or a double bond, e.g., compounds **16** and **17**, respectively, were inactive towards either cell lines. In contrast, an allyl or a 1-acetoxypropyl substituent at C(2), as in compounds **15** and **19**, respectively, increased the cytotoxicity moderately, compared to juglone (**1**). The greatest inhibitory effects were exhibited by compound **18** featuring a 1-acetoxyhomoprenyl substituent at C(2) like shikonin acetate (**4**). Interestingly, both enantiomers (**18a**-**b**) of compound **18** were almost as active as the racemic mixture on both cell lines (Table 1). As regards the contribution of a substituent at C(3) to the inhibitory effects of juglone methyl ethers **20** and **21**, the geranyl sidechain slightly increased the activity towards the H460 cells, compared to juglone (**1**).

Future research will be directed to the preparation of other 2-substituted juglone derivatives, including different esters of 2-(1-hydroxy-4-methylpent-3-en-2-yl) juglone, and to performing their MTT test on various human cell lines to explore possible selective antiproliferative effects.

## 4. Conclusions

The straightforward syntheses of naphthoquinones **12**, **15**, **16**, **17**, **18**, **19**, **20**, and **21** nicely illustrate two efficient procedures for the regioselective synthesis from non-symmetric starting compounds of juglone derivatives substituted at C(2) or C(3) by an aliphatic group. The methods outlined in this paper are, in principle, readily extendable to the preparation of other bioactive juglone derivatives for studies on the relationship between structure and bioactivity. In fact, an MTT test has indicated that an aliphatic substituent on the juglone nucleus significantly affects the inhibition of human cancer cell viability, in analogy with the cytotoxic properties of a few shikonin (**3**) and alkannin (**7**) derivatives.

More generally, we believe that the synthetic strategies implemented in this work shall pave the way towards the ready preparation of a wide variety of bioactive compounds based on the juglone nucleus.

## Figures and Tables

**Table 1:** In vitro inhibitory effects, expressed as IC_50_ (μM/L), of compounds **1**, **3**, **15**–**21** on the viability of H460 and MCF-7 cancer cells.

Tested Compound	H460 IC_50_ (μM/L)	MCF-7 IC_50_ (μM/L)	Tested Compound	H460 IC_50_ (μM/L)	MCF-7 IC_50_ (μM/L)	Tested Compound	H460 IC_50_ (μM/L)	MCF-7 IC_50_ (μM/L)
**1**	17.2	26.04	**17**	>25	>30	**19**	13.1	18.5
**3**	5.7	5.8	(±)-**18**	9.0	13.0	**20**	23.4	ND *
**15**	14.0	22.2	**18a**	9.2	13.0	**21**	11.4	ND *
**16**	>25	>30	**18b**	9.5	13.2			

* Not determined.

## Data Availability

Data is available from the authors.

## Supplementary Materials

The following supporting information can be downloaded at: https://www.mdpi.com/article/10.3390/biom15121708/s1, Figures S1–S9: ^1^H-NMR, ^13^C-NMR, and DEPT spectra of compounds **18**, **20**, and **21**; Figure S10–S12: Charge population in species A-C derived from deprotonation at C(3), C(2), and C(6), respectively, of 1,4,5-trimethoxynaphthalene (**25**); Figure S13: HPLC chromatogram of the resolution of compound (±)-**18**; Table S1: computed Cartesian coordinates of compound **25**.
