# Supplementary material for: Regioselective Synthesis and Cytotoxic Effects of New Juglone Derivatives with an Aliphatic Substituent at C(2) or C(3)
Source: Biomolecules. 2025 Dec 6;15(12):1708. doi: 10.3390/biom15121708 (PMC12730835; doi:10.3390/biom15121708)
Supplement: Supplementary file 1 [file biomolecules-15-01708-s001.zip › biomolecules-3981771-supplementary.pdf]

Supporting Material

Regioselective Synthesis and Cytotoxic Effects of New Juglone Derivatives with an Aliphatic Substituent at C(2) or C(3)

Giovanni Vidari,* Emanuele Casali, Andrea Magni, Sirwan T. Othman, Giuseppe Zanoni, Alessio Porta*

Table of Contents: page

Figure S1. ^{1}H-NMR spectrum of compound **18**. 2

. **Figure S2.** ^{13}C-NMR spectrum of compound **18**. 3

. **Figure S3**. ^{13}C-DEPT NMR spectrum of compound **18**. 4

. **Figure S4.** ^{1}H-NMR spectrum of compound **20**. 5

. **Figure S5.** ^{13}C-NMR spectrum of compound **20**. 6

. **Figure S6**. ^{13}C-DEPT NMR spectrum of compound **20**. 7

. **Figure S7.** ^{1}H-NMR spectrum of compound **21**. 8

. **Figure S8.** ^{13}C-NMR spectrum of compound **21**. 9

. **Figure S9**. ^{13}C-DEPT NMR spectrum of compound **21**. 10

. **Figure S10-S12**. Charge population in species A-C resulting from deprotonation at C(3), C(2), and C(6), respectively, of 1,4,6-trimethoxynaphthalene (**25**) with *n*-BuLi 11-13

Figure S13. HPLC chromatogram of the resolution of compound **18**. 14

Table S1. Computational output (Cartesian coordinates) of compound **25**. 15

.

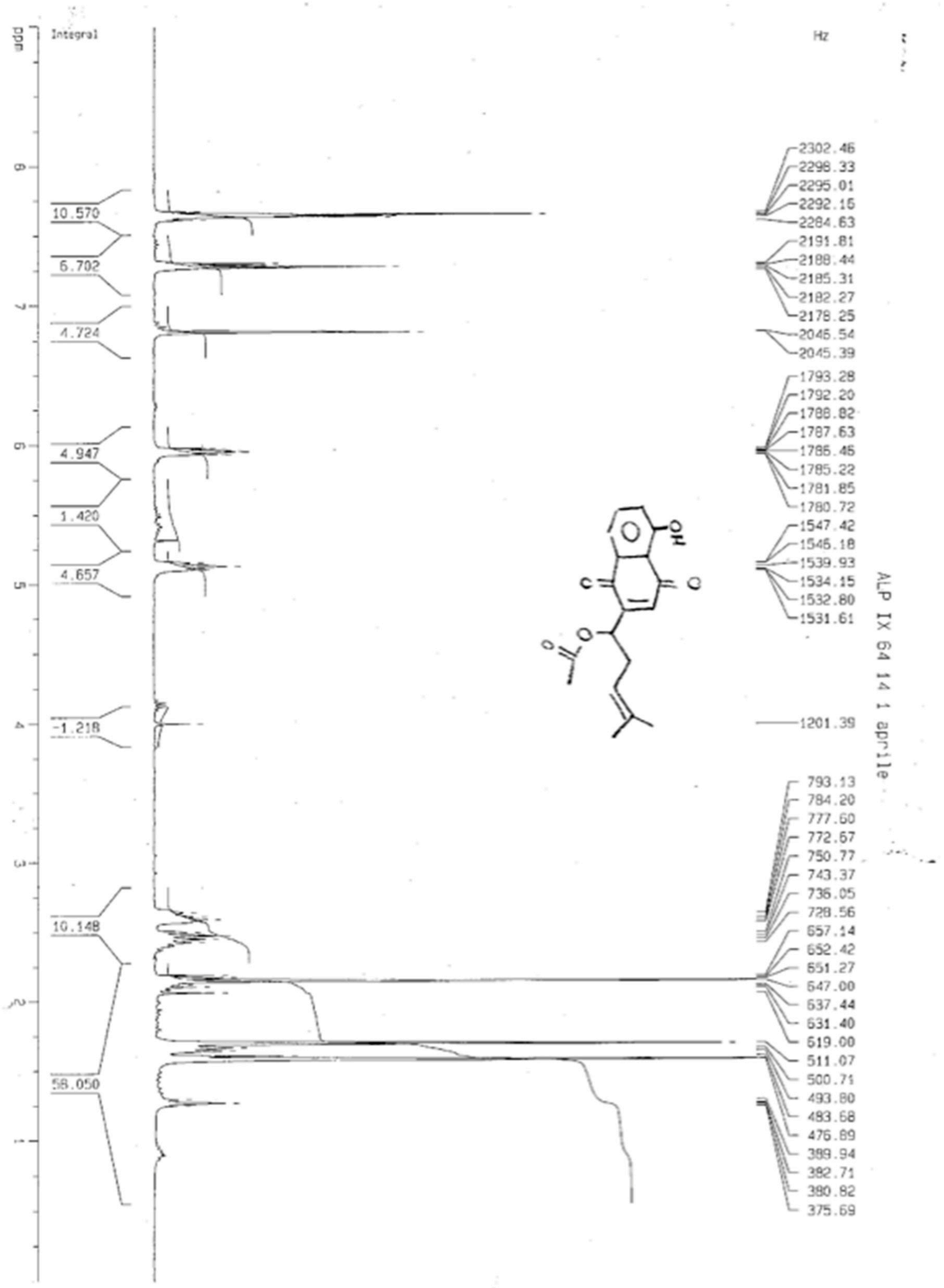

Figure S1. ^{1}H-NMR spectrum of compound **18**.

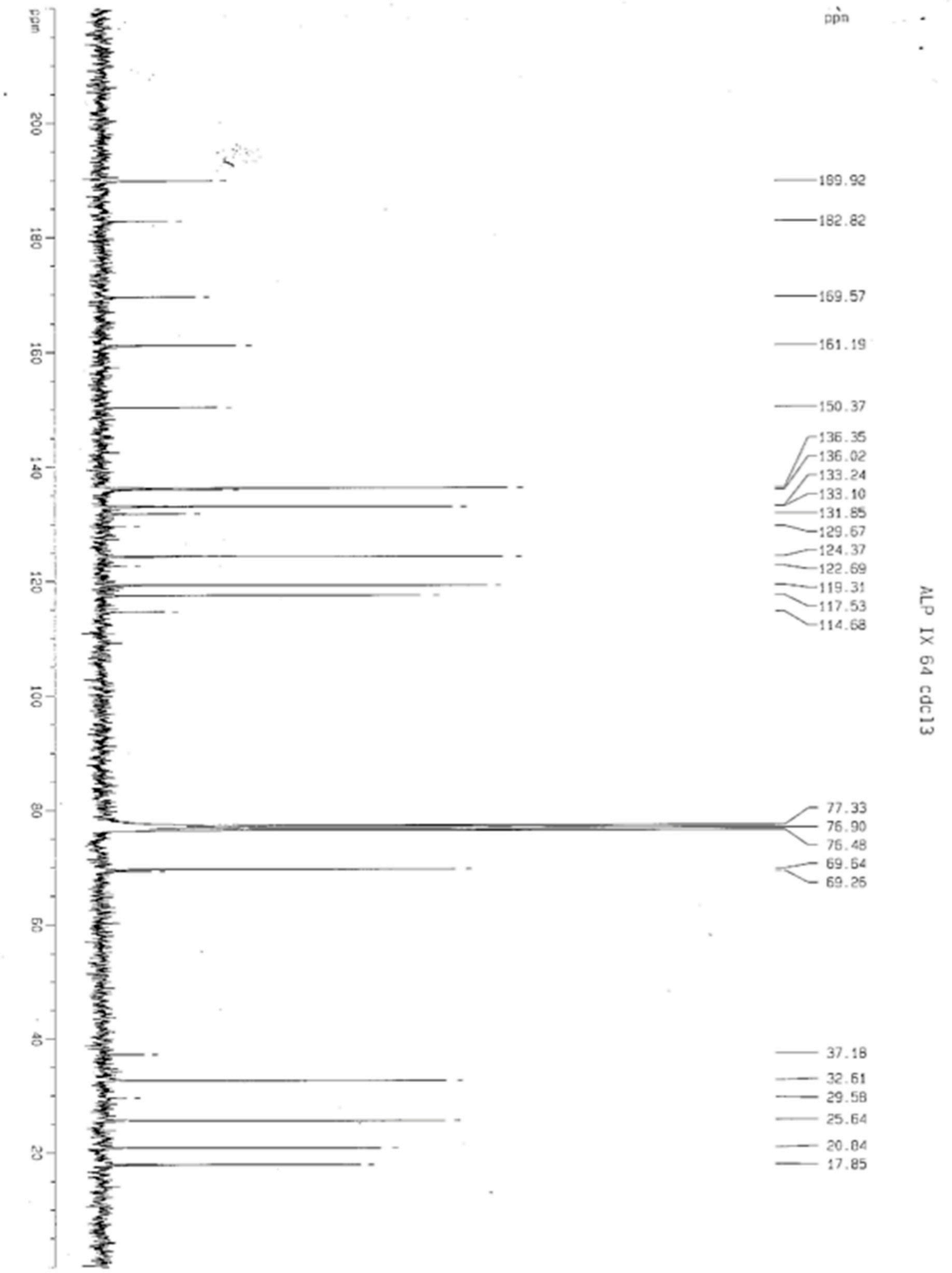

Figure S2. ^{13}C-NMR spectrum of compound **18**.

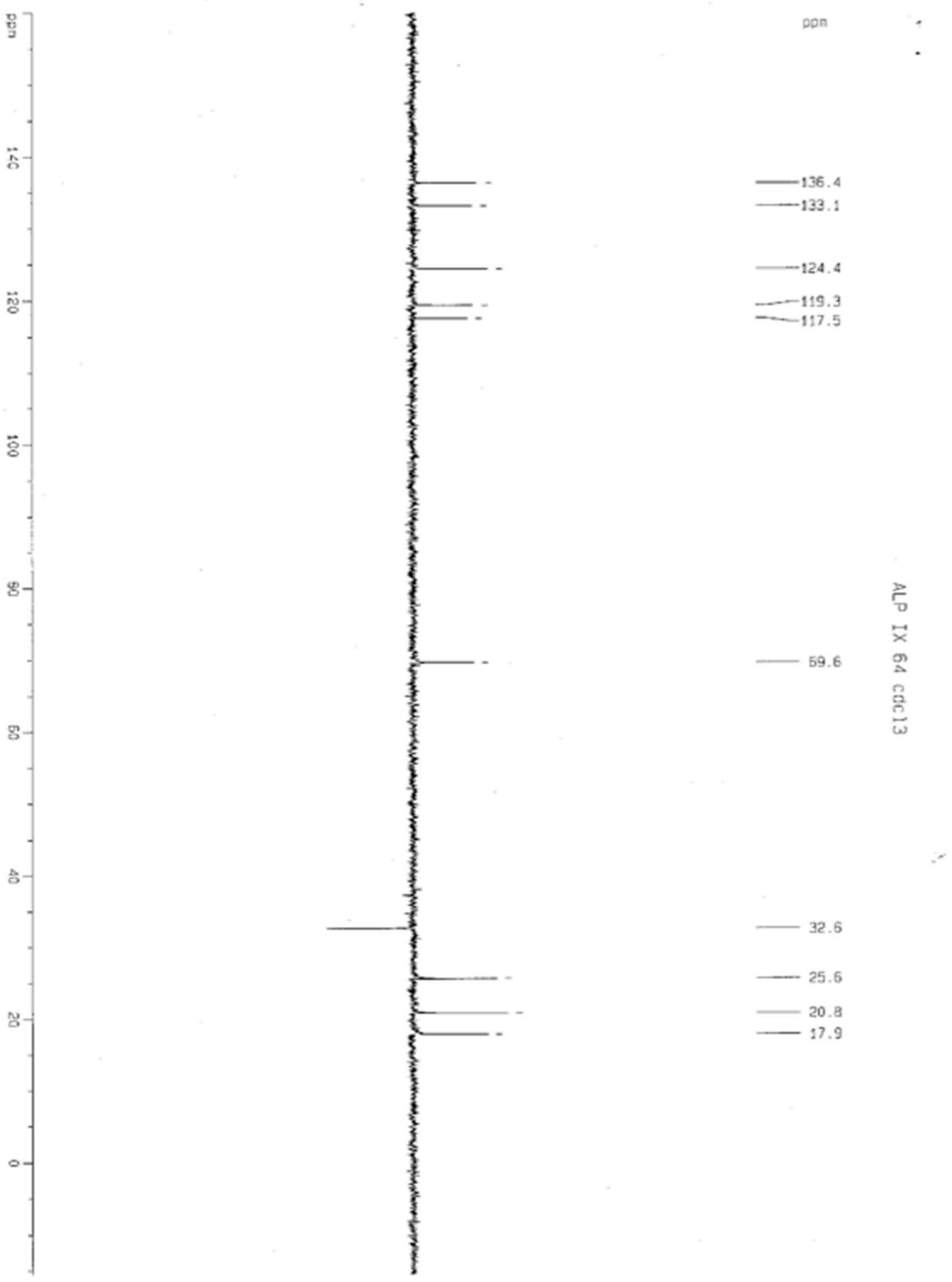

Figure S3. ^{13}C-DEPT NMR spectrum of compound **18**.

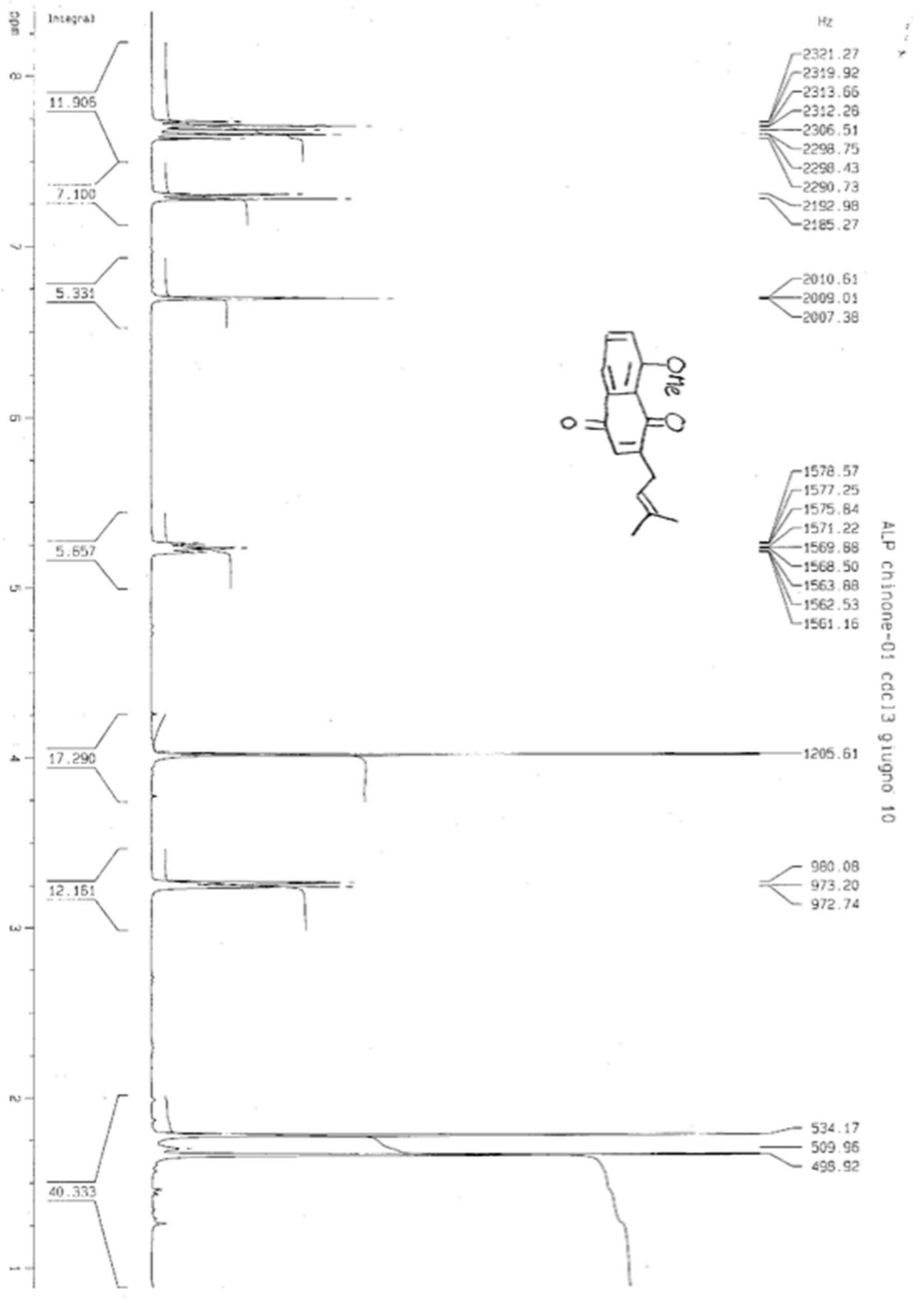

Figure S4. ^{1}H-NMR spectrum of compound **20**.

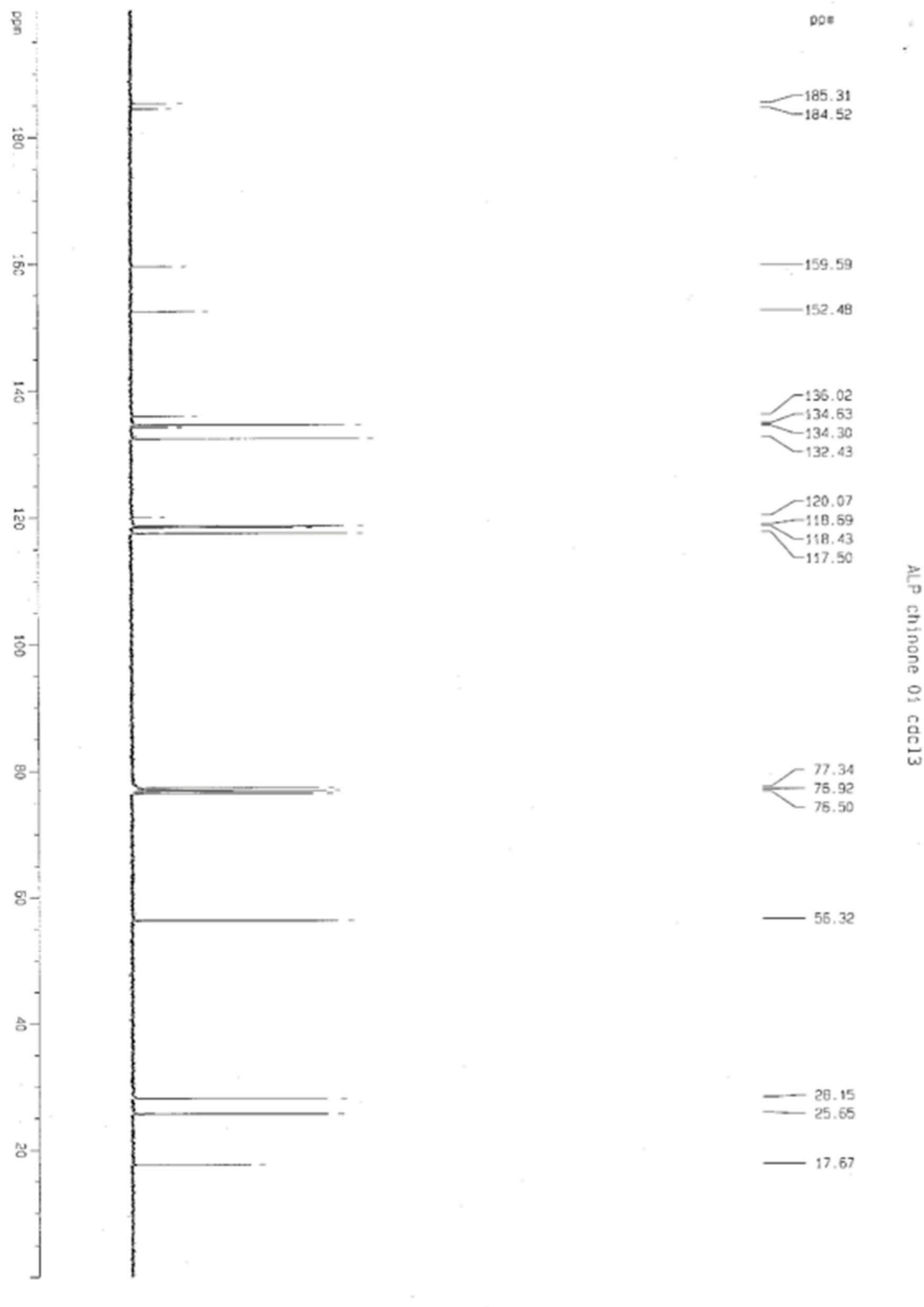

Figure S5. ^{13}C-NMR spectrum of compound **20**.

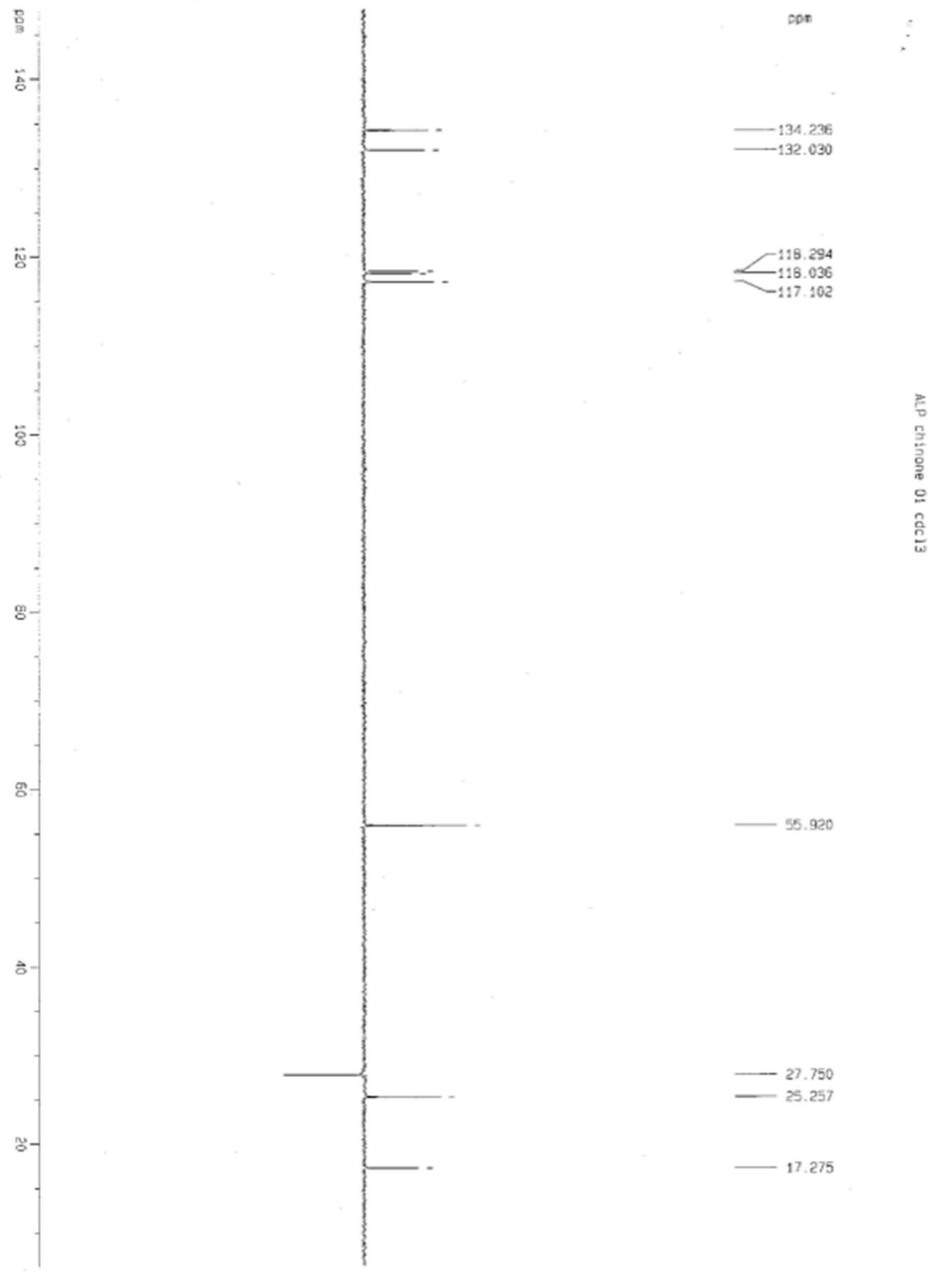

Figure S6. ^{13}C-DEPT NMR spectrum of compound **20**.

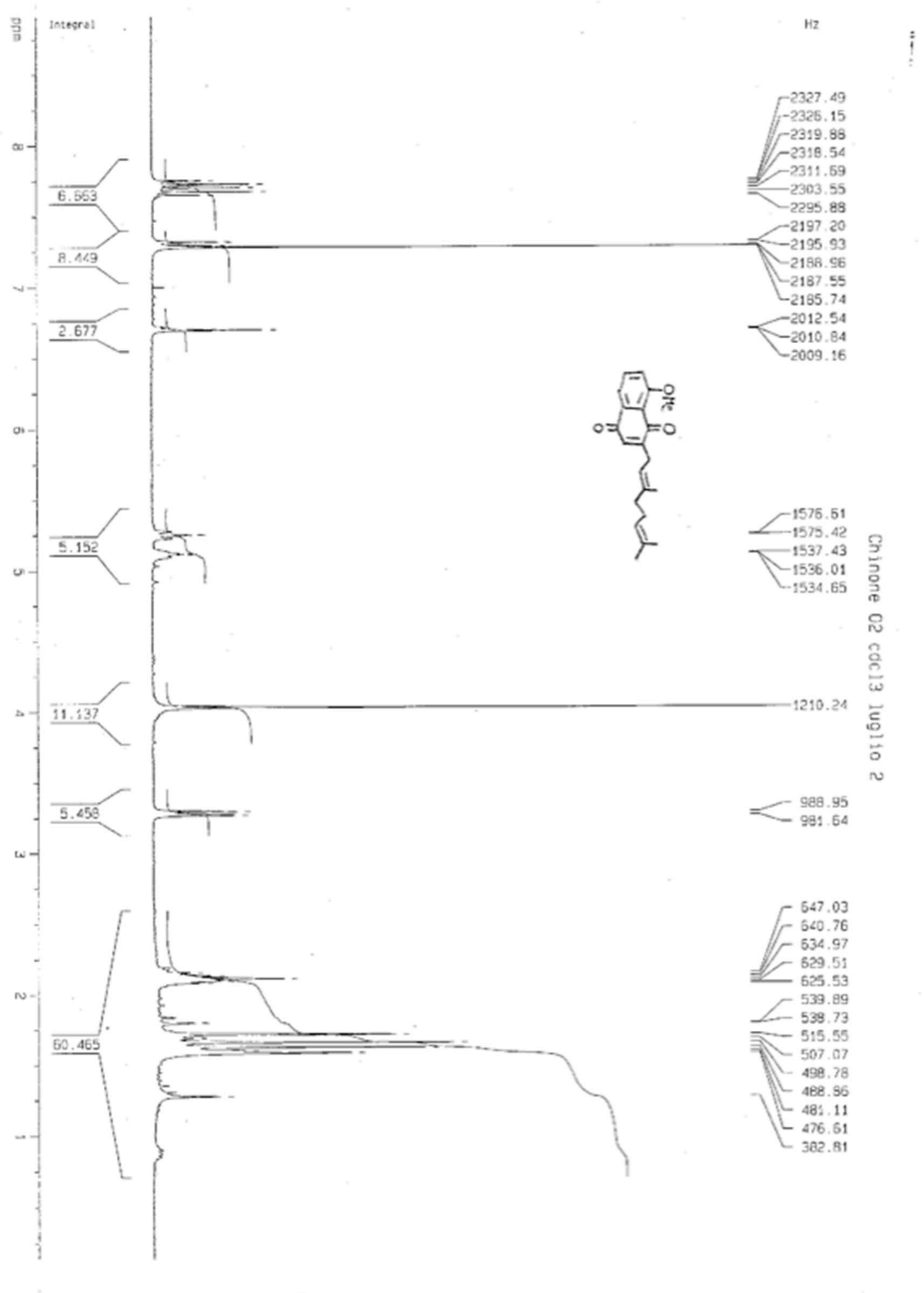

Figure S7. ^{1}H-NMR spectrum of compound **21**.

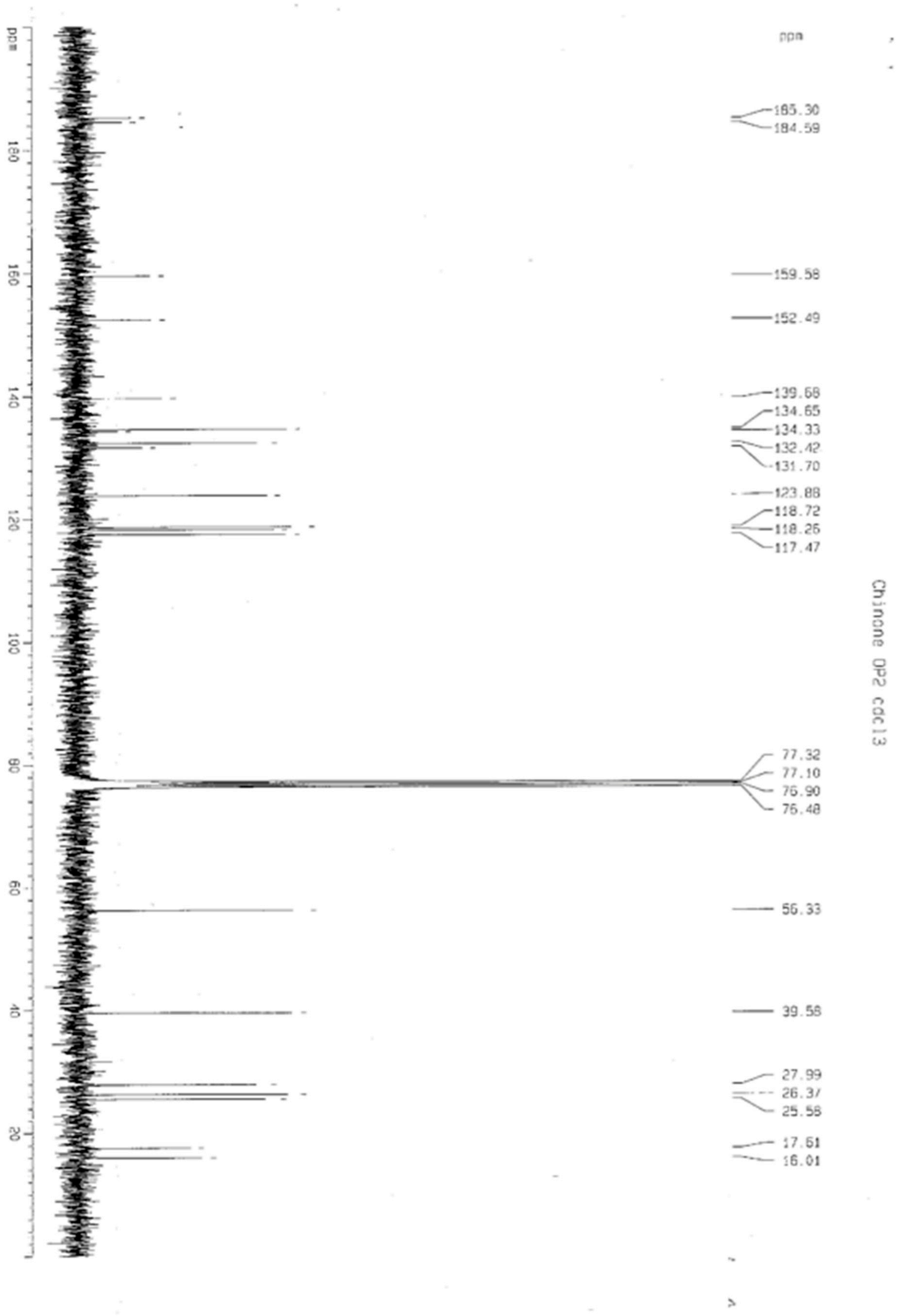

Figure S8. ^{13}C-NMR spectrum of compound **21**.

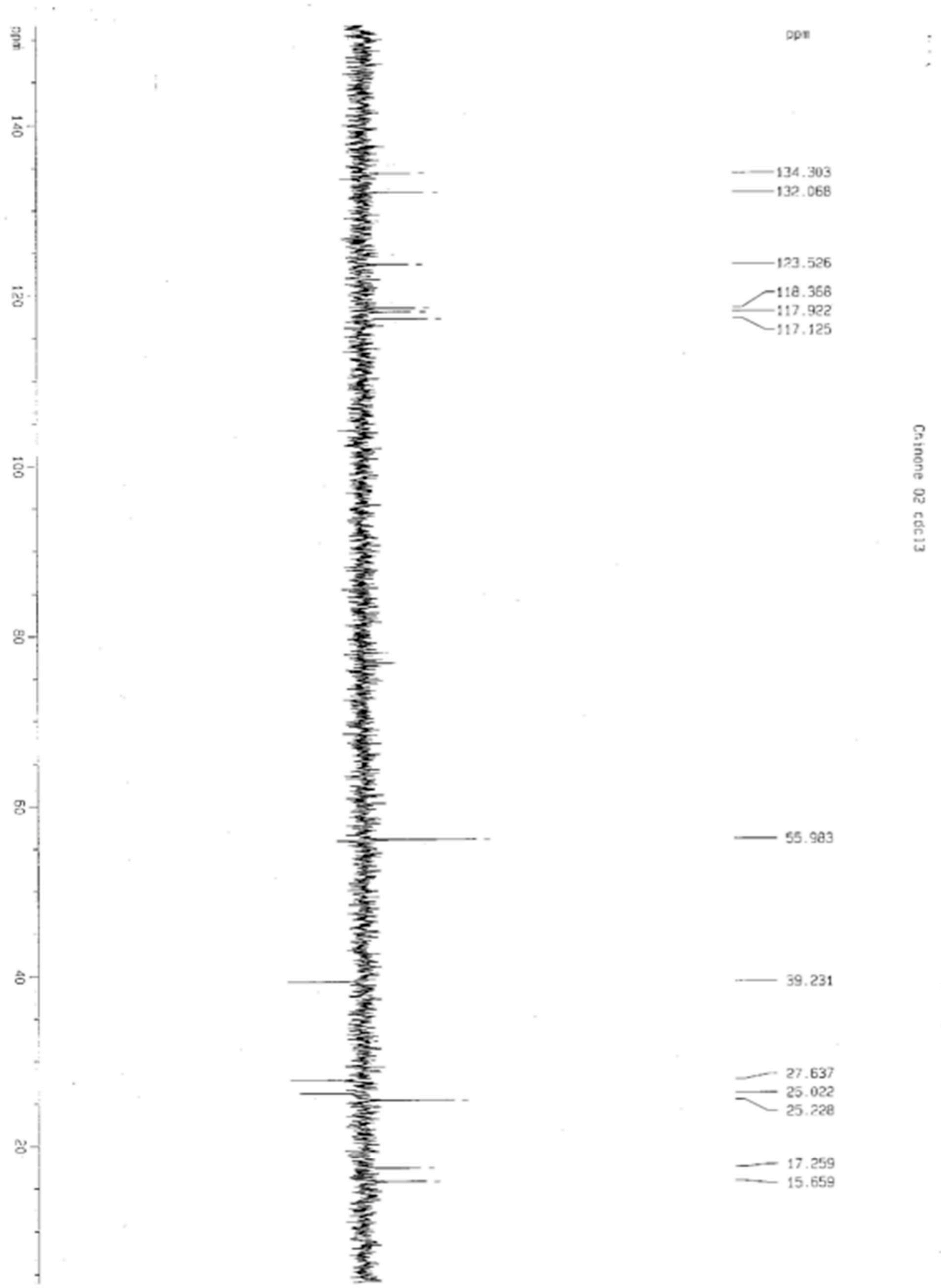

Figure S9. ^{13}C-DEPT NMR spectrum of compound **21**.

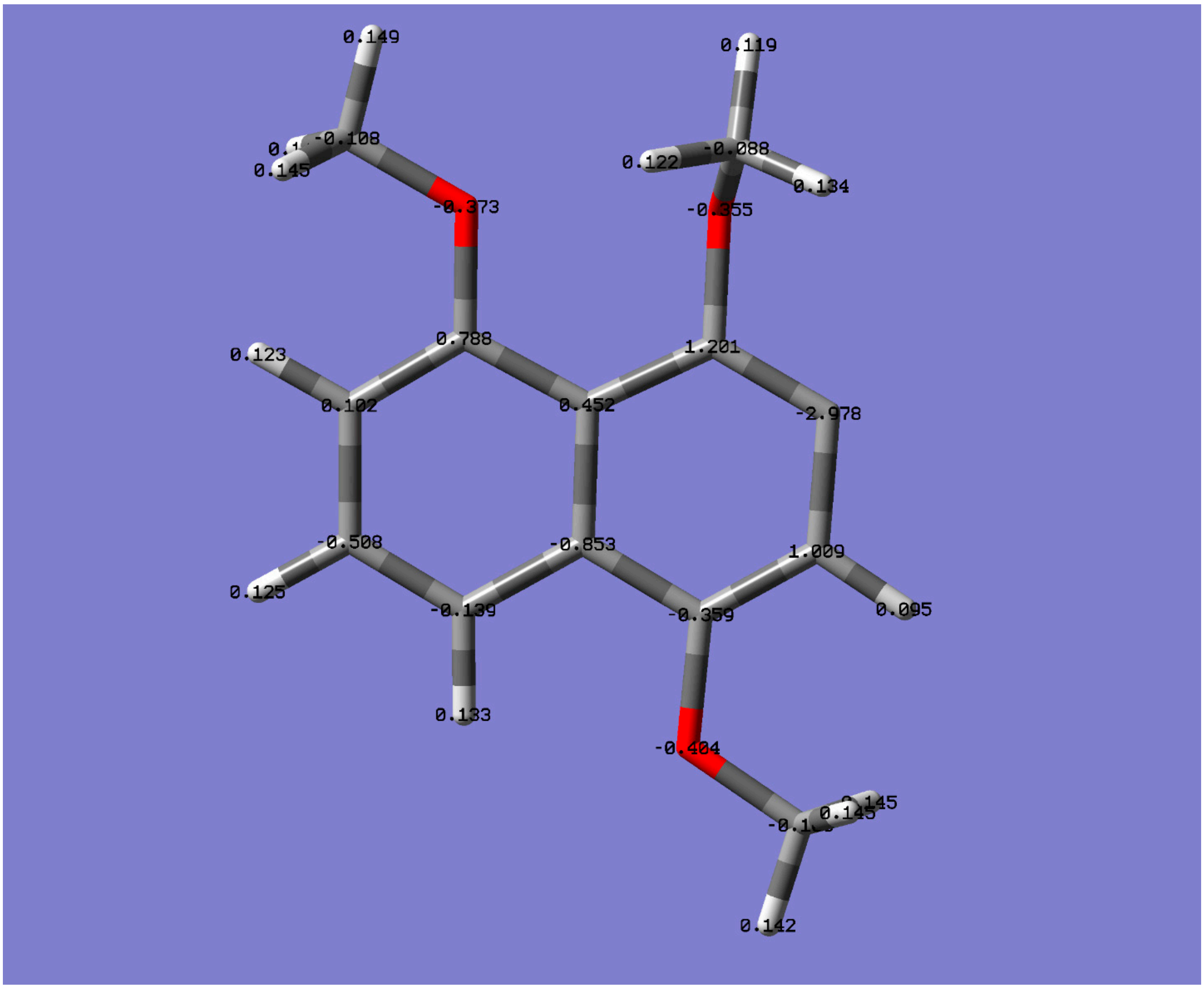

Figure S10. Charge population in species **A** resulting from deprotonation at C(3) of 1,4,5-trimethoxynaphthalene (**25**) with *n*-BuLi.

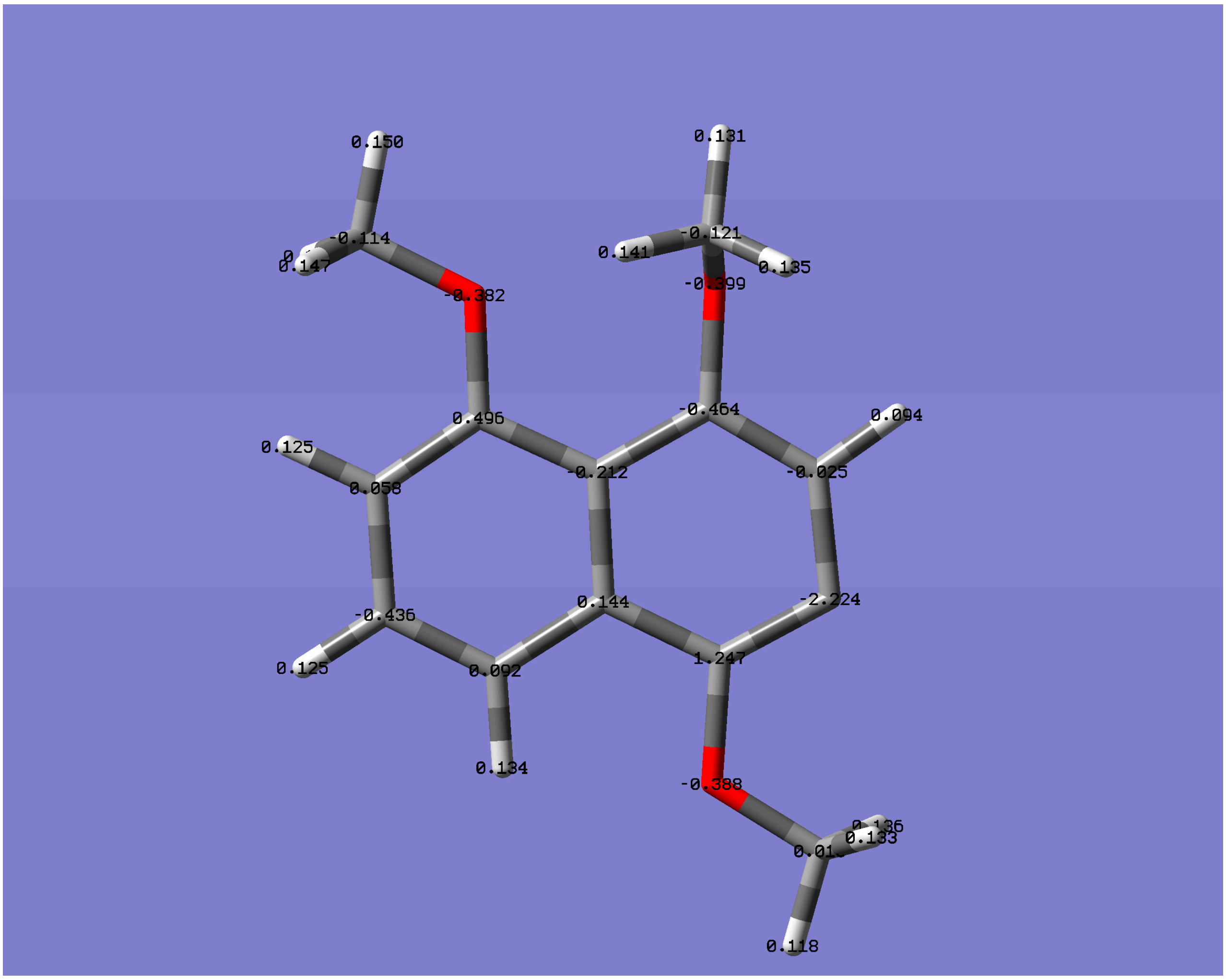

Figure S11. Charge population in species **B** resulting from deprotonation at C(2) of 1,4,5-trimethoxynaphthalene (**25**) with *n*-BuLi.

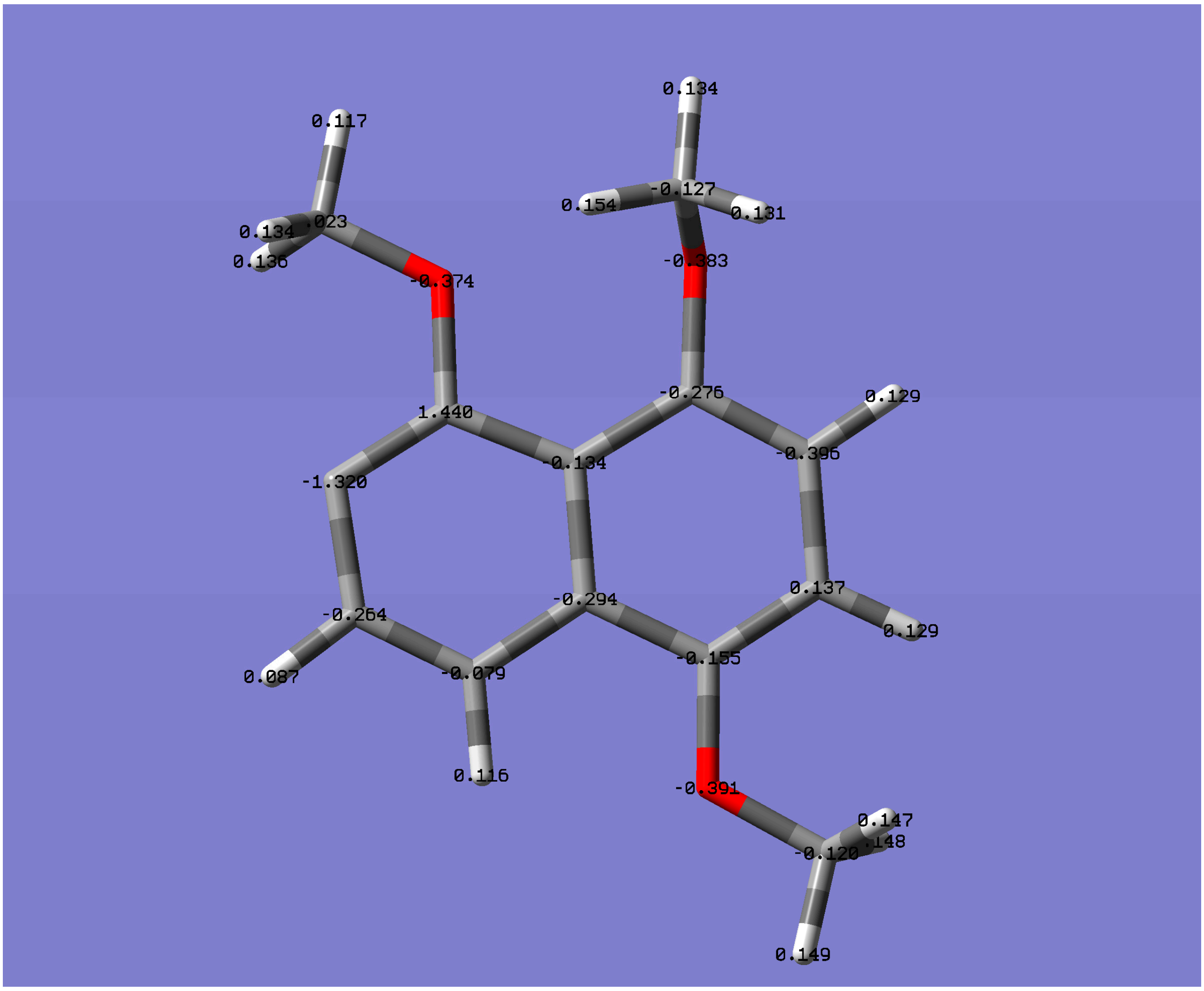

Figure S12. Charge population in species **C** resulting from deprotonation at C(6) of 1,4,5-trimethoxynaphthalene (**25**) with *n*-BuLi.

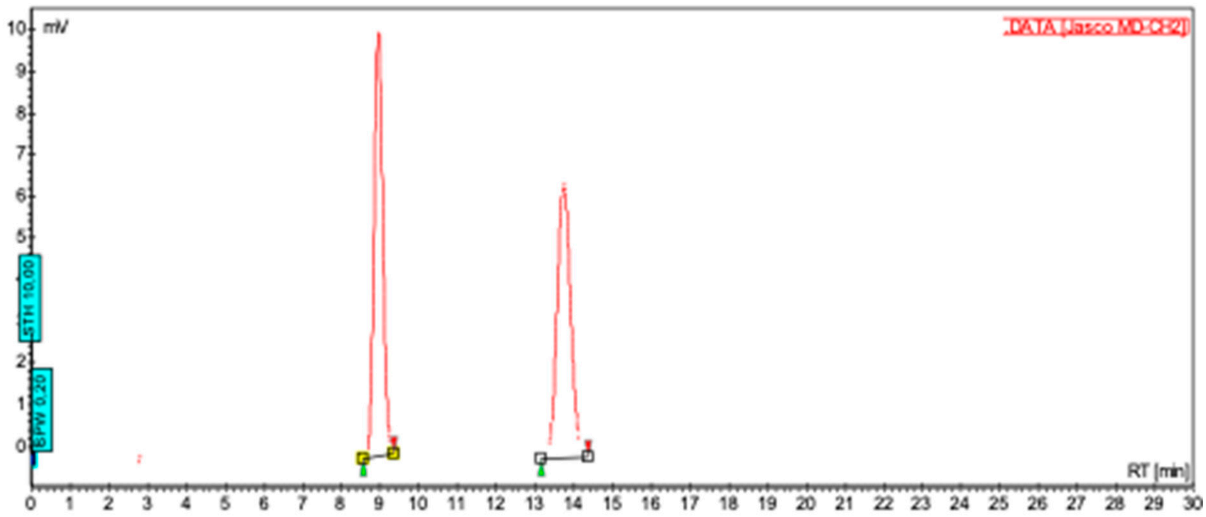

16a **16b**

Figure S13. HPLC chromatogram of the resolution of compound **18**.

Table S1. Computational output (Cartesian coordinates) of compound **25**.

C	-1.45420100	-2.20345300	0.05378800
C	-0.19829000	-2.83769000	0.16751400
C	0.96371700	-2.10242500	0.14577400
C	0.91271000	-0.68842400	0.00925200
C	-0.35265500	-0.00967900	-0.10228200
C	-1.54138600	-0.82616700	-0.07957000
C	2.13060500	0.07063000	-0.02539600
C	2.10296400	1.44208300	-0.17333900
C	0.85964600	2.10258000	-0.28038700
C	-0.33746000	1.42039400	-0.24283300
O	-2.73439000	-0.17690800	-0.19514900
C	-3.94008600	-0.94071000	-0.17170400
O	-1.49299400	2.16455900	-0.40427900
C	-2.11063900	2.59379000	0.81573900
O	3.27631500	-0.66651400	0.09153000
C	4.52944000	0.01725800	0.06450500
H	-2.35048300	-2.80980100	0.07095700
H	-0.16093400	-3.91821400	0.27031700
H	1.92870700	-2.58605500	0.22961900
H	3.01435700	2.02558100	-0.21026100
H	-4.05239200	-1.47911100	0.77642000
H	-3.97730500	-1.65147900	-1.00514200
H	-4.74705100	-0.21514400	-0.27660700
H	-2.43673700	1.73592000	1.41286100
H	-2.98073300	3.18797600	0.52939800
H	-1.42003300	3.21443800	1.40040200
H	5.29058900	-0.75526100	0.17523900
H	4.60799400	0.73124200	0.89235600
H	4.67375100	0.54066000	-0.88758500
H	0.83263400	3.18102600	-0.40116200
